# Cancer Stem Cells in Ovarian Cancer—A Source of Tumor Success and a Challenging Target for Novel Therapies

**DOI:** 10.3390/ijms23052496

**Published:** 2022-02-24

**Authors:** Jacek R Wilczyński, Miłosz Wilczyński, Edyta Paradowska

**Affiliations:** 1 Department of Gynecological Surgery and Gynecological Oncology, Medical University of Lodz, 4 Kosciuszki Str., 90-419 Lodz, Poland; 2 Department of Gynecological, Endoscopic and Oncological Surgery, Polish Mother’s Health Center—Research Institute, 281/289 Rzgowska Str., 93-338 Lodz, Poland; jrwil@wp.pl; 3 Department of Surgical and Endoscopic Gynecology, Medical University of Lodz, 4 Kosciuszki Str., 90-419 Lodz, Poland; 4 Laboratory of Virology, Institute of Medical Biology of the Polish Academy of Sciences, 106 Lodowa Str., 93-232 Lodz, Poland; eparadow@cbm.pan.pl

**Keywords:** cancer stem cells, ovarian cancer stem cells, ovarian cancer, therapy

## Abstract

Ovarian cancer is the most lethal neoplasm of the female genital organs. Despite indisputable progress in the treatment of ovarian cancer, the problems of chemo-resistance and recurrent disease are the main obstacles for successful therapy. One of the main reasons for this is the presence of a specific cell population of cancer stem cells. The aim of this review is to show the most contemporary knowledge concerning the biology of ovarian cancer stem cells (OCSCs) and their impact on chemo-resistance and prognosis in ovarian cancer patients, as well as to present the treatment options targeted exclusively on the OCSCs. The review presents data concerning the role of cancer stem cells in general and then concentrates on OCSCs. The surface and intracellular OCSCs markers and their meaning both for cancer biology and clinical prognosis, signaling pathways specifically activated in OCSCs, the genetic and epigenetic regulation of OCSCs function including the recent studies on the non-coding RNA regulation, cooperation between OCSCs and the tumor microenvironment (ovarian cancer niche) including very specific environment such as ascites fluid, the role of shear stress, autophagy and metabolic changes for the function of OCSCs, and finally mechanisms of OCSCs escape from immune surveillance, are described and discussed extensively. The possibilities of anti-OCSCs therapy both in experimental settings and in clinical trials are presented, including the recent II phase clinical trials and immunotherapy. OCSCs are a unique population of cancer cells showing a great plasticity, self-renewal potential and resistance against anti-cancer treatment. They are responsible for the progression and recurrence of the tumor. Several completed and ongoing clinical trials have tested different anti-OCSCs drugs which, however, have shown unsatisfactory efficacy in most cases. We propose a novel approach to ovarian cancer diagnosis and therapy.

## 1. Introduction

Ovarian cancer (OC) is the most lethal tumor of the female genital tract due to aggressive behavior, late diagnosis and high recurrence potential. Most of the patients worldwide are admitted with advanced disease as the initial steps of cancer growth are usually clinically obscured. This is a reason why the 5-year survival in the whole patient population does not exceed 48% (data of American Cancer Society 2020. https://www.cancer.org/cancer/ovarian-cancer/detection-diagnosis-staging/survival-rates.html, accessed on 20 December 2021). Moreover, ovarian cancer shows chemoresistance to standard platinum-based chemotherapy especially in advanced and recurrent cases, the fact which further influences poor survival. Ovarian cancer disease includes a heterogenous group of neoplasia: among them, about 90% are epithelial (subtypes: mucinous, serous, endometrioid and clear cells), as suggested by several and recent morphological and ultrastructural studies [1]. Ovarian cancer is a heterogeneous disease which comprise malignant tumors of serous, mucinous, endometrial or clear cell histology. According to the differences of biological behavior and malignancy, OC has been divided into two types: type I tumors containing low-grade (LGOC) serous, mucinous and endometroid ovarian cancer with better prognosis and lower rate mortality, and type II highly malignant and rapidly progressing high-grade serous ovarian cancer (HGOC) with poor prognosis and mortality about 90% of all OC cases [2,3]. Genetic expression profiling studies support this clinical classification, as type I tumors are associated with relative genetic stability and mutations of *PIK3CA*, *PTEN*, *BRAF*, *KRAS* and *ARID1A* genes, while type II tumors possess high chromosomal instability, defective homologous recombination repair and are characterized mostly by *TP53* mutations, but also by *BRCA1*, *BRCA2*, *RB1* and *CTNNB1* gene mutations [4,5,6,7,8,9]. Progenitor cells for type I OC are endometrial epithelial cells (for endometroid and clear cell tumors), tubal-peritoneal junction cells (for mucinous tumor) or fallopian epithelial cells and cortical inclusion cyst (CIC) epithelial cells (for LGOC), whereas for type II OC the progenitor cell originate from serous tubal intraepithelial cancer lesions (STIC) localized on tubal fimbriae. Early type I tumors frequently exist as so-called borderline tumors which do not show histologic signs of stromal invasion [1,2]. Recent gene profiling studies allowed for a proposal of a new classification based on both gene expression pattern and histological structure. According to this classification ovarian cancer could be divided into five subtypes: mesenchymal, immunoreactive, proliferative, differentiated and anti-mesenchymal. Mesenchymal and proliferative tumors comprise for 28% and 20% of OC, respectively. Mesenchymal subtype show desmoplasia and mesenchymal gene expression pattern, proliferative subtype show limited inflammatory infiltration and activation of signaling pathways for stemness. Both subtypes have an unfavorable prognosis. Otherwise, immunoreactive and anti-mesenchymal subtypes which comprise 21% and 12% of OC, have a better prognosis. The immunoreactive subtype is characterized by extensive T cell tumor infiltration and T-cell receptor and toll-like receptor signaling, while the anti-mesenchymal subtype shows a genotype which is opposite to the mesenchymal type. Differentiated subtype observed in 17% of OC tumors has gene pattern resembling serous borderline tumors and intermediate prognosis [10,11,12]. Extensive surgical debulking followed by platinum and taxane-based chemotherapy is a standard of care for invasive OC patients, however, extensive spread of tumor implants inside the peritoneal cavity, as well as a primary chemo-refractoriness or acquired chemoresistance of the tumor are responsible for unfavorable outcome. Recent studies suggest that a unique population of tumor cells called cancer stem cells (CSCs) are the most probable reason for cancer progression and therapy failure in OC.

## 2. Cancer Stem Cells—General Information

Cancer stem cells are a population of cells enable to reproduce the original phenotype of the tumor and capable of self-renewal, and due to those two properties they are crucial to tumor proliferation, differentiation, recurrence, metastasis, and chemoresistance [4,5,6,7,8]. The interactions between CSCs and immune cell populations in the immunosuppressive tumor microenvironment enable escape from immune surveillance and tumor development. CSCs were initially discovered in 1997 in acute myeloid leukemia and then identified in many solid tumors including prostate, ovarian, breast, pancreatic, colon, head and neck, lung, liver cancer and glioblastoma [13,14,15]. The CSCs abundance inside tumors could vary from 0.0001–0.1% to as many as 25% of tumor mass depending on the method of their identification, tumor environment, and origin from primary or recurrent/metastatic tumors [16,17,18]. The population of CSCs may be divided into two subpopulations: proliferating and quiescent CSCs occupying different niches inside the tumor. Proliferative CSCs are chemo-resistant but could be killed by over-standard doses of drugs, while quiescent CSCs are in an autophagic state and are capable to survive even high doses of chemotherapeutic drugs, thus promoting tumor relapse [13,19,20,21,22,23]. CSCs originate either through differentiation from progenitor or normal stem cells or from normal cancer cells which acquire stemness via the epithelial-mesenchymal transition (EMT) process [21,24]. Nowadays an EMT process is viewed as a continuum of states from a fully epithelial/proliferative to fully mesenchymal/invasive phenotype comprising a spectrum of intermediate hybrid states. CSCs could represent any of these phenotypic states showing outstanding plasticity [25]. According to the CSCs properties there are two models of tumor growth functioning. The hierarchical model points out that CSCs are the only population exhibiting a self-renewal capacity. According to the alternative stochastic model, all cancer cells are capable to transform into CSCs and undergo self-renewal or differentiation into non-proliferating cancer cells. A situation met in tumors seems to be rather a mixture of what is described by pure hierarchical and stochastic models [26,27]. This plasticity model takes into account a flexible and dynamic understanding of the tumor niche with the goals of immunosuppression and therapeutic evasion. It depends on cancer cells genotype, as well as epigenetic and environmental signals from tumor microenvironment (TME) [13]. Stressors (hypoxia, pH, drugs, mechanical stress, immunological response), stress-triggered epigenetic changes (i.e., histone and non-coding RNA modifications) and activation of “stemness” signaling pathways (i.e., wingless-related integration site—Wnt, Hedgehog, neurogenic locus notch homolog protein—NOTCH) regulate CSCs. In solid tumors different areas of the tumor possess different stressors intensity which could influence both phenotype and function of CSCs [28,29,30,31,32,33,34,35]. This is another background for the observed outstanding plasticity of CSCs. Moreover, analysis of the genomic profile of ovarian cancer CSCs indicated that stemness is rather a functional state than an attribute of a particular cell type [36]. Therefore, targeting the management against CSCs would be a great challenge.

The problem of CSCs is directly connected to the phenomenon of cancer dormancy, defined clinically as cancer systemic or local recurrence after a long time in a patient who has been considered as completely cured and free of the disease. On the cellular level cancer dormancy is dependent on the existence of cells possessing partially overlapping functions and belonging to populations of circulating tumor cells (CTCs), disseminated tumor cells (DTCs) and CSCs. CTCs are a population of tumor cells shed into circulation and moving towards the potential metastatic sites. Their number correlates with tumor aggressiveness. CTCs with high metastatic potential might represent a population of circulating CSCs [37]. DTCs represent a population of dormant tumor cells residing inside target organs, mainly in bone marrow, showing a prolonged growth arrest and living inside microenvironmental niches. Their population could be enriched by CSCs [38]. The population of quiescent CSCs is considered to participate in cancer dormancy due to slow cellular division and extraordinary resistance to microenvironmental stressors like hypoxia, tissue acidity and metabolic starvation. Quiescent CSCs and dormant DTCs show over-expression of signaling pathways responsible for adaptation to hypoxia (hypoxia-induced transcription factor-1α—HIF-1α; glucose transporter-1—GLUT1), activation of dormancy (nuclear receptor subfamily-2 group-F member-1—NR2F1) and survival in a hostile environment (mammalian target for rapamycin—mTOR) [39,40,41]. CTCs, DTCs and CSCs are cell populations capable of producing micro metastases (disseminating both from early-stage small tumors and late-stage advanced tumors). These cells migrate towards target organs where they reside in pre-metastatic niches which are actively created by both cancer cells and local cells recruited from stroma (cancer-associated fibroblasts—CAFs, myeloid-derived stem cells—MDSCs) and immune system (tumor-associated macrophages—TAMs, T regulatory lymphocytes—Tregs) [42,43,44,45]. In the pre-metastatic niche DTCs/CSCs wait until the moment when signals from the local environment change the niche into the mature metastatic niche. The signal triggering this transformation is frequently a result of inflammation and activates angiogenic pathways (“angiogenic switch”) to initiate metastatic growth of dormant tumor [46,47]

## 3. Ovarian Cancer Stem Cells (OCSCs)—Markers

### 3.1. Cell Surface Markers

CSCs surface markers are not specific as they are also expressed on normal stem cells. Therefore, CSCs should also be identified by precisely defined behavior, such as spheroid formation or the reconstitution of tumors after transplantation to laboratory animals. Numerous markers have been suggested to identify CSCs, including OCSCs; however, their precise clinical significance is still unknown. Despite this, several surface cell markers identifying OCSCs isolated either from patient samples or experimental animals and cancer cell lines have been described (Table 1).

#### 3.1.1. Glycoprotein CD44

Molecule CD44 is a cell-surface glycoprotein that is a receptor for the hyaluronic acid receptor. Its stimulation activates signaling pathways including epidermal growth factor receptor (EGFR)/Ras small GTPase protein (Ras)/extracellular signal-regulated kinase (ERK) and transcription factor homeobox protein NANOG-dependent signaling pathways, followed by cell proliferation, differentiation, and increased motility and stemness. Interaction between NANOG and STAT3 results in the up-regulation of multidrug resistance protein-1 (MDR1) and the effective efflux of chemotherapeutic drugs from OCSCs [48,49]. The population of CD44+ OC cells possess self-renewal, tumor initiating and sphere-forming capacities. Recurrent OC shows a higher expression of CD44-positive cells compared to primary tumors which is correlated with poor prognosis, although not all studies are consistent in this matter. CD44 exists in alternatively spliced variants which could be better correlated to the clinical history of OC. Between them CD44v6 was found in excess on OCSCs from distant metastases indicating metastasis-initiating activity via a hematogenous spread. In patients with FIGO stage I-III OC, distant metastasis-free survival was better in patients with CD44v6-low tumors. Silencing of the CD44-regulatory gene with siRNA resulted in tumor shrinkage in a mouse model of OC [49,50,51,52,53,54,55,56,57,58].

#### 3.1.2. Receptor Tyrosine Kinase CD117

CD117 is receptor tyrosine kinase coded by c-kit proto-oncogene. It has an external ligand-binding domain specific for a stem cell factor (SCF) and a cytoplasmic domain with intrinsic tyrosine kinase activity. Binding CD117 to SCF causes the receptor proteins to dimerize, phosphorylation and activation. This activates several signaling pathways, mainly Ras/ERK, phosphoinositide-3-kinase (PI3K)/protein kinase-B (AKT), non-receptor tyrosine kinase Src and Janus kinase (JAK)/signal transducer and activator of transcription (STAT), responsible for regulating cell proliferation, differentiation, apoptosis and adhesion. CD117 identifies a population of sphere-forming non-adherent OC cells and a so-called “side population” of OC cells having the capacity to harbor a specific position on flow-cytometric panels due to the selective expression of ATP-binding cassette drug transporters (ABC membrane transporters) using Hoechst 33,342 dye-staining. The basis of the “side-population” assay is the ability of ABC transporters to provide a rapid efflux of lipophilic fluorescent dye, and they are identified using special gating strategies during the analysis of flow-cytometric plots. Since its first use for hematopoietic stem cell identification, the “side-population” assay has been successfully used for the identification of progenitor and stem cells in different tissues, including stem-like cancer cells displaying increased capacity of self-renewal, tumorigenicity and chemo-resistance [59]. The “side population” shows an increased expression of stemness genes. OC cells bearing both CD44 and CD117 markers are abundant in chemo-resistant OCSCs [55,58,60,61,62,63,64,65]. The presence of CD117+ OC cells are correlated with resistance to standard platinum-taxane-based chemotherapy and shorter recurrence intervals in treated OC patients, while the inhibition of CD117 results in the restitution of chemo-sensitivity. The level of CD117 expression correlates with both disease-free and overall survival. The expression of CD117 could be augmented by hypoxia and HIF-1α activation, and is followed by Wnt/β-catenin signaling [63,66,67]. SCF growth factor is expressed in high concentrations in the ascitic effusions collected from EOC patients. The soluble SCF form is produced by TAMs and fibroblasts (TAFs), whereas a minority of tumor cells only express the membrane-associated SCF form. C-kit is expressed by OCSCs and binds to both soluble and cell-associated SCF, as well as functionally responding to the ligand [68].

#### 3.1.3. Prominin-1 CD133

Surface protein CD133 is also called prominin-1 and is a transmembrane glycoprotein activating the PI3K/Akt pathway. It is responsible for tumor maintenance, vascularization, and chemoresistance, and hence is considered to be an OCSC marker. It was found that CD133 mediates metastatic homing to peritoneal tissue in OC [69]. Endothelin receptor-A (ETRA) is expressed on CD133+ cells and the chemotherapy-induced inhibition of ETRA decreases the ability of CD133+ cells to form spheres [70]. The activity of nuclear factor-κ-light chain enhancer of activated B cells (NF-κB) and p38 MAPK pathways in response to IL-17 enhances the self-renewal capabilities of CD133+ cells. The expression of intracellular stemness markers octamer-binding transcription factor-4 (OCT4) and sex-determining region Y—box 2 (SOX2) is higher in CD133+ than CD133- cells [71]. Ovarian C-X-C chemokine receptor type-4 (CXCR4)+/CD133+ OC cells showed more effective platinum efflux and lower platinum sensitivity compared to CD133-negative cells [72]. The correlation between CD133 expression and advancement of the tumor (presence of HGOC, advanced clinical stage, presence of ascites, tumor non-responsive to chemotherapy), as well as patients’ survival has been pointed out [73]. Moreover, the population of CD133+ cells was more abundant in recurrent platinum-resistant tumors compared to primary OC tumors [74]. However, according to some studies, OCSCs indicate an inconsistent expression of the CD133 marker and CD133+ cells do not always have particular pro-tumorigenic properties. It is possible that OCSCs plasticity and some heterogeneity could account for this controversy. Another possibility is an alternating expression of the CD133 molecule in cytosolic and membrane compartments of OCSCs populations [75,76,77,78,79,80,81,82,83,84].

#### 3.1.4. Heat-Stable Antigen CD24

CD24 is a transmembrane adhesion molecule also called a heat-stable antigen CD24. Through the activation of the STAT3 signaling pathway, it is able to stimulate cell adhesion and augment cell malignancy. The inhibition of the JAK2/STAT3 pathway in OC results in a loss of cancer stemness and reduced tumor growth [85]. The inhibition of the over-expressed JAK2/STAT3 pathway in patient-derived CD24+ OCSCs correlated with a better survival of OC patients [86]. The CD24 molecule may also regulate NANOG and OCT4 expression, thus stimulating tumor growth and resistance to chemotherapy. CD24-positive OC cells were isolated both from tissue specimens and from cellular cultures, and were shown to overcome anoikis and to form spheroid structures. CD24+ OC cells also displayed the up-regulation of genes regulating cellular stemness. The abundance of CD24+ cells was increased in metastatic peritoneal implants compared to the primary tumor, where it contributed to the attachment of OC cells to fibronectin and collagen of the peritoneal stroma. STAT3 signaling following CD24 stimulation is a well-established stimulator of stemness of OCSCs cells, while PI3K/AKT and mitogen-activated protein kinase (MAPK) signaling following CD24 stimulation could activate EMT. However, there are also scanty opinions that CD24 expression has no correlation with OCSCs’ self-renewal and chemoresistance [87,88,89,90,91,92,93,94,95]. Clinically, high CD24+ expression is a predictor of poor survival in OC patients [96].

#### 3.1.5. MyD88 Protein

Myeloid differentiation primary response gene 88 (MyD88) is an adaptor protein for signals generated by toll-like receptor-type 4 (TLR-4). TLR-dependent signaling activates NF-κB which moderates the inflammatory pathway in tumor carcinogenesis. The TLR-4/MyD88 pathway has been modified in OC and is responsible for OC chemo-resistance. MyD88-positive ovarian cancer cells equate to OCSCs cells due to the resistance to pro-apoptotic signals and the ability to create a pro-inflammatory tumor microenvironment through leukocyte recruitment. MyD88-negative cells are more differentiated and less aggressive. The expression of MyD88 protein was found to be an unfavorable prognostic factor for OC patients [97,98].

#### 3.1.6. Epithelial Cell Adhesion Molecule EpCAM

Epithelial cell adhesion molecule (EpCAM) is a type I transmembrane glycoprotein regulating intercellular adhesion, present on a subset of normal epithelia, as suggested by several recent studies [99]. EpCAM-positive OC cells have greater tumor-initiating potential compared to EpCAM-negative cells. The EpCAM/B-cell lymphoma-2 (Bcl-2) signaling pathway prevents platinum-dependent apoptosis of cancer cells resulting in chemo-resistance; therefore, EpCAM expression is increased in tumors of chemo-resistant patients and correlates with unfavorable outcome [100]. Similarly, the B-cell lymphoma extra-large (Bcl-xL) anti-apoptotic protein present in mitochondria was found to be over-expressed in recurrent chemo-resistant OC. The inhibition of Bcl-xL restored chemo-sensitivity of OC cells [101].

#### 3.1.7. Multipositivity of Cell Surface Markers

Effective isolation of OCSCs usually demands the identification of two or more cell markers. Double positive CD44+/CD117+ cells are highly capable to recapitulate the original tumor after being transplanted into experimental animals, and are the main component of sphere-forming cells in ascites. This OCSC population also showed high mitochondrial concentrations of reactive oxygen species (ROS) suggesting that mitochondrial respiration is used to sustain OCSC’s viability in stress conditions and during starvation [102]. The level of CD44+CD24-OCSCs in OC patients has been suggested to have a prognostic value. In patients having more than 25% of CD44+CD24-OCSCs, a greater probability of recurrence and a shorter progression-free survival were observed. Similarly, primary tumors showed either a low or high expression of CD44+ALDH1+ OCSCs. Those exhibiting low expression had a better response to chemotherapy and longer progression-free survival [103,104,105,106]. Recurrent platinum-resistant ovarian tumors compared to primary tumors are enriched in the population of CD44+CD133+ALDH1A1+ OCSCs. The population of CD44+/E-cadherin-/CD34- inside ovarian tumors identify OCSCs cells with the ability to recapitulate the tumor and support neovascularization [107]. The population of CD44+CD166+ has stem cell characteristics through the increased capacity of forming spheres and the high activity of histone deacetylases regulating the OCSC’s phenotype [108]. CD133+/ALDH1+ cells have tumor initiating properties and induce neoangiogenesis. CD44+/MyD88+ cells are highly resistant against apoptosis and chemotherapeutic drugs. They can grow as adherent cells or are able to undergo EMT and form spheroid cell structures [109]. Similarly, CD44+/CD24+/EpCAM+ cells show OCSCs’ properties having increased migratory and invasive potential and chemo-resistance to platinum, taxane and doxorubicin [110]. Cells of CXCR4+CD133+ phenotype isolated from OC cell lines also have OCSC properties [77]. Neoadjuvant chemotherapy is a therapeutic option for patients in whom primary optimal cytoreductive surgery is unavailable due to extensive peritoneal carcinomatosis. However, studies have revealed that this form of management is associated with the enrichment of metastatic tumors in OCSCs defined as ALDH1+ cells showing chemo-resistance and correlated with bad prognosis [111,112]. Recent studies have shown that not only standard platinum-taxane-based chemotherapy contributes to the proliferation of OCSC population; targeted therapy with poly-(ADP) ribose polymerase (PARP) inhibitors which disturb tumor DNA repair systems also result in an enrichment of tumors with OCSCs followed by a restored ability to repair DNA [58].

The variegated opinions about OCSC markers could have several origins. The simplest explanation is that different OCSC populations are characterized by different markers. Another possibility is that due to ovarian cancer histological and genetic heterogeneity, the observed populations of OCSCs follow this heterogenic pattern. Moreover, OCSCs could differ between distinct localizations and in different stages of the disease. Finally, the marker diversity could result from OCSCs’ molecular and functional plasticity, where cells with different properties share stemness and tumor propagating abilities [113,114]. In this context, patients could have multiple populations of OCSCs which may vary between distinct tumors and patients. This possibility creates an unfavorable perspective for the success of therapy directed against OCSCs.

### 3.2. Intracellular and Functional Markers

#### 3.2.1. Aldehyde Dehydrogenases ALDH

Aldehyde dehydrogenases (ALDH) are a group of enzymes converting aldehyde substrates to carboxylic acids via the oxidation process. The protective and detoxifying function of the ALDH1 subgroup is involved in the maintenance of cancer cells, especially OCSCs, against chemotherapeutics and radiation. In this subgroup, the most supporting role for the creation of the OCSC phenotype is assigned to ALDH1A1 and ALDH1A2 isotypes [115]. ALDH1-positive cell phenotype identifies the OCSC populations possessing self-renewal and stemness properties that are capable of sphere formation and restoring the tumor. ALDH1 exerts its function via the Wnt/β-catenin signaling pathway and ABC-transporters. ALDH1 activity is also regulated by NF-κB/transcription factor RelB non-canonical pathway [115]. Moreover, ALDH1 is engaged in the activation of OCSCs quiescence program by slowing down the proliferation of the cells and in the protection of DNA-repair programs that both contribute to ovarian cancer resistance. It was found that primary OC tumors contain less ALDH1+ cells compared to tumors pre-treated with neoadjuvant chemotherapy [111,116,117,118,119,120,121,122,123]. The observation that, in cultures of OC cells, the percentage of ALDH1+ cells was growing along with the dose escalation of platinum was a very alarming phenomenon, indicating great viability and endurance of this cell population [81]. Epidermal growth factor-like domain-6 (EGFL6), which functions as a stem cell regulatory factor activates an asymmetric division of ALDH1+ OC cells stimulating their proliferation [106,124,125]. In HGOC patients, tumors characterized by the higher percentage of double-positive ALDH+/epidermal growth factor receptor (EGFR) + cells, are associated with poor outcomes as compared with tumors that are either ALDH or EGFR negative [126]. In clear-cell carcinoma population of ALDH1^high^ OCSCs, cells show markedly higher tumorigenic potential than ALDH1^low^ cells. The expression of ALDH1^high^ OCSCs is increased in advanced tumors and correlates with unfavorable prognosis and chemo-resistance [106,120,127].

#### 3.2.2. Transcription Factors

A group of transcription factors have also been considered to be markers of OCSCs. These include NANOG, SOX2, forkhead-box protein M1 (FOXM1), and OCT4. NANOG is physiologically responsible for the maintenance of self-renewal and the pluripotency of embryonic stem cells (ESCs), the same role it plays in OC, where it additionally regulates EMT and chemo-resistance via the STAT3 signaling pathway. The expression of NANOG in OCSCs cells correlates with clinical stage and high grade, as well as resistance to standard chemotherapy [50]. Similar to NANOG, SOX2 is also responsible for the maintenance of self-renewal and the pluripotency of ESCs. The over-expression of SOX2 is related to the stemness of cells via the inhibition of the PI3K/AKT pathway, resulting in resistance to apoptosis. In OC, pathological SOX2-positive cells were identified in the epithelium of the tubal fimbriae of patients with HGOC tumors and patients with germline *BRCA1/2* mutations [128,129]. OCT4 is involved in embryonic development and cellular pluripotency. Its function is to stabilize the higher-order structure of chromatin in the NANOG locus [130]. Up-regulation of OCT4 in OCSCs was correlated to tumor initiation and again, chemo-resistance. The cytoplasmic expression of OCT4 regulates EMT transformation and is a recognized predictor of adverse clinical outcomes in cancer. The co-expression of OCT4 with RNA-binding protein Lin28 was connected to advanced tumor growth and grade [131]. Increased levels of OCT4 were observed in OCSCs’ CD24-positive cells [88]. NANOG, OCT4 and SOX2 were over-expressed not only in tumor tissues but also in both ascites and spheres built from OCSCs cells [129,132,133]. FOXM1 is a member of the FOX family of transcription factors. It plays an important role in cell cycle control and progression, and in the maintenance of genomic stability. The over-expression of FOXM1 protein was observed in OCSCs exposed to elevated concentrations of lysophosphatidic acid (LPA) present in ascites fluid in OC patients. Increased FOXM1 levels were followed by the activation of wingless and Int-1 (Wnt)/β-catenin signaling and chemo-resistance. Alternatively, FOXM1 suppression resulted in the restitution of chemo-sensitivity and the loss of ability to spheroid formation in the peritoneal environment [134,135,136].

The cells characterized by OCSC phenotypes were identified both in ovarian surface epithelium (OSE) and fallopian tube epithelium (FTE) in mice, including ALDH1+, ALDH1A1+, ALDH1A2+, CD133+ and NANOG+ cells. These OCSCs markers were detected especially in the distal portion of the tube (fimbria) supporting the observation of HGOC origin [137,138].

**Table 1:** Markers of OCSCs—function, correlation to clinicopathological features and their cell/tissue origin.

Marker	Function	Origin of Studied Cells	Reference	Association to Clinicopathological Features	Cell/Tissue Origin	Reference
CD44+	Increased tumorigenicity, sphere-formation, cells self-renewal	Primary EOC tumors, cell cultures	[49,50,51,52]	Number of CD44+ cells higher in early stage EOC and correlated with shorter PFS Expression correlated with high-grade, advanced (III/IV FIGO) EOC in younger (<60) patients Higher number of CD44+ cells correlated with chemoresistance and shorter DFI CD44+ correlated with Ki67 index, p53 positivity and tumor grade in HGSOC, mucinous and endometroid EOC	EOC-isolated cells Recurrent EOC (88% HGSOC) Primary and recurrent EOC (78% HGSOC) EOC (HGSOC 62%) and BOT	[105,139,140,141]
CD44 v6+	Increased tumorigenicity, recapitulation of tumors	Xenotransplantation model	[57]	Distant metastases more frequent and metastasis free survival shorter in CD44v6+—high group of patients Increased number of CD44v6+ cells in primary tumors correlated with shorter OS	EOC FIGO I–III tumors EOC FIGO III–IV tumors (71% HGSOC)	[56,57]
CD44+/MyD88+	Increased tumorigenicity, sphere-formation, resistance to apoptosis, chemoresistance	Cell lines, ascites	[142]	Expression of MyD88 protein was an unfavorable prognostic factor for EOC patients	Benign ovarian tumors, BOT and EOC (54% HGSOC)	[97]
CD44+/CD117+	Increased tumorigenicity, sphere-formation, recapitulation of tumors, chemoresistance	EOC tumors, xenograft models	[49]	CD44+CD117+ cell lines were less prone to paclitaxel-induced apoptosis	EOC cell lines	[142]
CD44+/CD24-	Increased tumorigenicity, sphere-formation	Cell lines	[143]	>25% CD44+/CD24- cells in ascites correlated with higher risk of recurrence and shorter PFS	Ascites-isolated cells from advanced EOC	[104]
CD44+/CD24+/ EpCAM+	Increased tumorigenicity, chemoresistance	Cell lines, EOC-isolated cell lines, ascites	[100,110]	Ovarian cancer stem cells expressing EpCAM+ are less prone to chemotherapy and are a source of recurrent tumor after the treatment	EOC I-IV FIGO stage (45% HGSOC, 14% clear cell, 17% endometroid, 12% mucinous)	[100]
CD44+/CD166+	Increased tumorigenicity, sphere-formation	Cell lines	[108]	Population of platinum-resistant cells is enriched in CD44+/CD166+ population	EOC-isolated and standard cell lines	[144]
CD44+ALDH1+	Increased tumorigenicity, chemoresistance	Cell lines	[145]	>50% ALDH1+ cells correlated with shorter OS	Advanced EOC (73% HGSOC)	[145]
CD44+/CD133+/ALDH1A1+	Chemoresistance	Cell lines, EOC-isolated cell lines	[116]	Expression of markers increased in recurrent compared to primary tumors	Advanced primary and recurrent EOC	[116]
CD133+	Increased tumorigenicity, enhanced vasculogenesis	Cell lines, EOC tumors, xenograft models, ascites	[72,78,81,146]	Expression of CD133+ correlated with presence of HGSOC, higher FIGO stage, ascites, chemoresistance, shorter PFS and OS No correlation with prognosis Expression of CD133+ correlated with shorter PFS and OS Expression of CD133+ correlated with shorter OS and platinum chemo-resistance	EOC (67% HGSOC) EOC FIGO III–IV (72% HGSOC) Advanced metastatic HGSOC Advanced primary HGSOC	[73,147,148,149]
CD133+/ALDH1A+	Increased tumorigenicity, cells self-renewal, chemoresistance	EOC tumors, cell lines, xenograft models	[80,81]	Expression of CD133+ correlated with III/IV FIGO stage, expression of CD133+/ALDH1A+ correlated with shorter PFS and OS	HGSOC	[150]
CD117+	Increased tumorigenicity, sphere-formation, recapitulation of tumors, chemoresistance	EOC-isolated cell lines, xenograft model, ascites	[62,63,64,151,152]	Expression of CD117+ correlated with shorter PFS 40% of HGSOC were CD117+ and expression correlated with chemoresistance	Advanced metastatic HGSOC HGSOC	[63,148]
CD24+	Increased tumorigenicity, stimulation of EMT	Cell lines	[92]	Expression of CD24+ correlated with FIG stage and the presence of peritoneal and lymph node metastases	27% HGSOC 12% mucinous 18% clear-cell 18% endometaroid 23% others	[92]

BOT—borderline ovarian tumor; DFI—disease-free interval; EMT—epithelial-mesenchymal transition; EOC—epithelial ovarian cancer; FIGO—International Federation of Obstetrics and Gynecology; HGSOC—high-grade serous ovarian cancer; OS—overall survival; PFS—progression-free survival.

## 4. Signaling Pathways in OCSCs

The activation of intracellular signaling pathways responsible for stemness is a key step for the survival of CSCs. The most important pathways engaged in CSCs’ function are Wnt/β-catenin, Hedgehog, Hippo/Yes-associated protein (YAP), NOTCH, NF-κB and HIF-1α. Wnt/β-catenin is a canonical and conservative signal pathway necessary for the initiation and regulation of cell self-renewal, growth, migration, survival and organogenesis. Hedgehog (Hh) signaling is extremely important for the interaction between CSCs and CAFs of tumor niche. The Hippo/YAP pathway is essential signaling for the regulation of tissue growth, organ size, and stemness maintenance. NOTCH signaling is a conservative cell-to-cell communication pathway responsible for cell proliferation, differentiation and tissue angiogenesis. NF-κB-signaling regulates multiple processes including proliferation, angiogenesis, migration and particularly inflammation. HIF-1α signaling is one of the key pathways important to perform cancer cell proliferation, EMT transition, dormancy and chemo-resistance in hypoxic conditions [153,154,155,156,157,158,159].

### 4.1. Wnt/β-Catenin Signaling Pathway

In ovarian cancer, CD117+ OCSCs up-regulate the Wnt/β-catenin signaling pathway, thus activating the program of chemo-resistance in a hypoxic environment probably by the up-regulation of ATP-binding cassette superfamily-G member 2 (ABCG2) transmembrane transporters. The knockdown of β-catenin reverses the expression of the ABCG2 transporter and restores platinum and taxane sensitivity [60,160]. The activation of the Wnt/β-catenin pathway in OCSCs is mediated by the activation of leucine-rich repeat containing G-protein coupled receptors-5 and -6 (LGR5 and LGR6) which are stemness markers [161]. The Wnt/β-catenin signaling is also an important regulator of EMT and OCSCs with high zinc-finger transcription factor SNAI1, and the (SNAIL)/E-cadherin ratio is more mobile and resistant to therapy [162]. The secreted frizzled-related protein-5 (SFRP5) which is a Wnt antagonist is frequently silenced in OC, and the restoration of SFRP5 function inhibits Wnt/β-catenin-dependent signals and EMT and abrogates chemo-sensitivity [163]. Moreover, the Wnt/β-catenin pathway is engaged in interactions between TAMs and CAFs promoting aggressive behavior and pro-inflammatory phenotypes of CAFs [164,165]. It was shown that ascites from highly invasive OC contain exosomes filled with micro-RNAs (miRNAs) targeting the Wnt/β-catenin pathway, and thus stimulate metastases [166,167]. When xenografted to mice, human platinum-resistant OC tumors showed the up-regulation of Wnt/β-catenin target genes (axis inhibition protein-2 gene—*AXIN2*, dickopf-related protein-2 gene—*DKK2*, *LGR5*) and showed an increased expression of OCSC markers (CD24, ALDH1, EpCAM) [135].

### 4.2. Hedgehog-Signaling

The activation of Hh-signaling in OCSCs augments chemo-resistance and stimulates the formation of spheroid structures [168,169,170]. Zinc-finger protein GLI1 and SMO (smoothened class frizzled G-protein coupled receptor) are effectors of Hh-signaling. The formation of spheroidal cell aggregates or spheroids possessing OCSC properties were correlated to increased GLI1 expression. Moreover, GLI1 enhances the ABCB1 and ABCG2 transporters in spheroid-forming OC cells making them chemo-resistant. Borderline, malignant, and platinum-resistant OC showed higher expressions of GLI1 and SMO compared to benign and chemo-sensitive tumors [169,170,171].

### 4.3. Hippo/YAP Pathway

Stiffness of extracellular matrix (ECM) and shear stress are, among others, signals that activate the Hippo/YAP pathway. These mechanosensory signals are important components of the OC environment in the peritoneal cavity, and up-regulation of Hippo/YAP signaling in OCSCs promotes cell proliferation, metastasis, and chemo-resistance in ovarian cancer [172].

### 4.4. NOTCH Pathway

About ¼ of OC tumors indicate the disturbed expression of genes responsible for NOTCH pathway regulation. Together with NOTCH overexpression, other components of the NOTCH-dependent pathway were up-regulated in OC including vascular-endothelial growth factor (VEGF), vascular-endothelial growth factor receptor-1 (VEGFR1), delta-like ligand-4 (DLL4) protein and cell surface protein Jagged1 (JAG1). Hypoxic conditions inside ovarian tumors promote the deregulation of both NOTCH and HIF-1α signaling, which enhances the stemness and migration capacities of OCSCs. NOTCH also simulates an expression of intracellular markers of OCSCs such as OCT4 and NANOG, as well as ATP-binding cassette superfamily-B member 1 (ABCB1), thus regulating platinum resistance. High activity of NOTCH3 was noticed especially in ALDH+ OCSCs and “side population” and correlated with paclitaxel-resistance. Overactivity of NOTCH2 and NOTCH3 was observed in advanced and recurrent OC compared to primary tumors, and correlated with short overall and disease-free survival. The inhibition of the NOTCH pathway significantly attenuates OCSC function, and restores sensitivity to drugs and the induction of apoptosis [124,153,168,173,174].

### 4.5. NF-κB Signaling

In ovarian cancer, BRCA1 mutation is constitutively associated with the activation of the NF-κB p65 subunit, and in OC treated with inhibitors of DNA damage repair, with activation of NF-κB p50 subunit, respectively. This NF-κB and BRCA1 interdependence is responsible for OC chemo-resistance. The inhibition of NF-κB signaling in cisplatin-resistant OC lines induces apoptosis and decreases the number of CD44+ OCSCs [175,176,177,178]. CAFs, which constitute one of the key components of the TME, stimulate overexpression of ECM proteoglycan versican which, through binding to the CD44 molecule, activates NF-κB and c-Jun N-terminal kinase (JNK) signaling in OCSCs [179,180]. Moreover, interleukin (IL)-17, which stimulates the creation of CD133+ cell spheres and OCSCs’ self-renewal, exerts its effects through NF-κB- and p38 mitogen-activated protein kinase (p38MAPK) pathways [71]. OCSCs are capable of autocrine stimulation by cytokines that activate inflammatory signaling pathways, thus contributing to tumor progression. CD133+ OCSCs stimulated by IL-23 enhance their self-renewal capabilities by the activation of NF-κB and STAT3 pathways. The activation by chemokine (C-C motif) ligand-5 (CCL5) and its receptors promotes the migration of CD133+ OCSCs and their differentiation into endothelial cells via the activation of NF-κB and metalloproteinase-9 (MMP-9) [181,182,183,184]. Constitutive NF-κB activity is attributed to CD44+MyD88+ OCSCs, and the activation of TLR/MyD88/NF-κB pathway correlated with increased numbers of OCSCs [70,185].

## 5. OCSCs and Tumor Microenvironment

### 5.1. CSCs Niche—General Considerations

CSCs niche is a cancer cell microenvironment that participates in the maturation and regulation of CSCs. Components of the niche provide both nutrients and signals indispensable for the proper function of CSCs. The niche is composed of CAFs, mesenchymal stem cells (MSCs), TAMs, tumor-infiltrating lymphocytes (TILs), non-CSCs cancer cells, adipocytes, components of extracellular matrix, vessels, inflammatory cytokines, and chemokines. The niche provides signals for CSC differentiation, supports CSCs’ resistance to apoptosis and toxic agents, and accumulates epigenetic signals [186,187]. One of the most important cellular components of CSCs niche are CAFs, which regulate EMT transition, secrete pro-angiogenic factors, produce cytokines (IL-6, leukemia inhibiting factor—LIF; transforming growth factor-β—TGFβ), chemokines (IL-8, (C-X-C) motif chemokine-12—CXCL12, CXCL1), prostaglandins (PGE) and growth factors (hepatic growth factor—HGF, VEGF) [188,189]. Mesenchymal stem cells (MSCs) migrate into sites of inflammation, tissue injury, and cancer where they suppress the immune response and participate in the regulation of EMT, angiogenesis, and chemo-resistance, and are able to differentiate into CAFs [190,191,192]. Cancer-associated adipocytes (CAAs) provide lipids for CSCs, which are stored inside as lipid droplets. High concentration of lipid droplets is correlated with tumor aggressiveness and poor survival. Fatty acids provided by CAAs serve as an energetic reserve for the CSCs during periods of starvation [193]. Lipid desaturation plays an important role in the self-renewal and tumorigenicity of CSCs through the changes in the lipid composition of the cell membrane and Wnt/β-catenin signaling [194]. ECM composition is altered inside the tumor niche and influences CSCs’ behavior, mainly EMT, hypoxia, and chemoresistance. Components of ECM could cooperate with CSCs to augment stemness and metastases [195]. CSCs can adapt to variable levels of tissue oxygenation inside tumors, and are capable of functioning using both aerobic glycolysis and oxidative phosphorylation (OXPHOS) [196]. Hypoxia-dependent HIF-1α activation is able to reprogram CSCs. HIF-1α enhances EMT and stemness activators such as Wnt/β-catenin, Hedgehog, NOTCH pathways and CD133, Nanog and Sox2 markers [197,198]. Acidosis of TME maintains CSCs’ stemness, activates the OXPHOS mechanism, and changes lipid metabolism and drug resistance [199]. The inflammation in TME is directly connected to EMT transition and up-regulates the resistance of CSCs against host immune surveillance. Several pro-inflammatory cytokines/chemokines, including TGF-β, tumor-necrosis factor- α (TNF-α), IL-1, IL-6 and IL-8 are secreted by the cells in the CSCs’ niche. Cytokine-triggered signaling pathways activate transcription factors and epigenetic regulation in CSCs [199].

### 5.2. OCSCs’ Niche in Ovarian Cancer

#### 5.2.1. Initiation and Growth of Primary OC Tumors

Investigations performed in mice identified two distinct microenvironments (niches) responsible for the initiation and growth of primary OC tumors. Niches contain progenitor stem cells which could evolve into OCSCs; however, knowledge about precise OCSC–TME interactions in those two niches is still scarce. The first one is the ovarian surface epithelial niche (OSE) localized in the hilum of the ovary in the junctional region between the ovarian surface, peritoneal mesothelium, and the fallopian tube. The OSE niche contains LGR5+ stem cells prone to neoplastic transformation caused by mutations in cellular tumor antigen p53 (*Trp53*) and retinoblastoma protein-1 (*RB1*) tumor suppressor genes [138]. The second niche is the fallopian tube epithelium (FTE) in the distal portion of the tube where fimbria is localized. A similar FTE niche could be found in humans. It contains CD44-expressing stem cells which could be subjected in vitro into immortalization and form HGOC, mimicking tumors in mice following xenotransplantation. Moreover, the FTE niche in women carriers of germline *BRCA* mutations contained increased numbers of SOX-2-expressing stem cells more abundant in HGOC patients compared to patients with benign ovarian tumors [200,201,202]. Significantly broader knowledge pertains to the OCSCs’ metastatic niches in the peritoneal environment (peritoneal mesothelium, omentum, ascitic fluid) and distant organs. In advanced, metastatic and recurrent OC, interactions between OCSCs and peritoneal TME seem to play a key role in tumor maintenance and chemoresistance, thus strongly contributing to therapy failure. Passive dissemination of OC inside the peritoneal cavity (transcoelomic spread) is a dominant way of production of metastases.

#### 5.2.2. Ascites

Ascites is a unique microenvironment for OCSCs, and accounts for the transcoelomic spread of metastases, also called peritoneal implants. Ascites also facilitates the entry of cancer cells into lymphatic vessels. Tumor cancer cells go through EMT, seed from the primary localization as cell spheroids enriched in OCSCs, and are transported passively with fluid into distant localizations in the peritoneal cavity, going through MET and starting to grow extensively [179,203]. OC cells undergoing EMT acquire OCSCs’ characteristics. Sphere-forming cells are shown to express CD117 and ALDH1, as well as NOTCH1, CUB domain-containing protein-1 (CDCP1), and NANOG [204]. They can resist anoikis, which normally eliminate cells without any anchorage to the background. Peritoneal disseminated tumors are also enriched in CD44v6-positive cells which may contribute to transcoelomic dissemination as they have increased tumor-initiating capability [57]. Zinc-finger transcription factor SNAI2 (SLUG), a repressor of E-cadherin, and SNAIL assure resistance to p53-mediated apoptosis in OCSCs cells during EMT [51]. Ascites contains soluble factors which enrich OCSC populations in both spheroids and implants, including IL-6, IL-8, IL-10, VEGF and osteoprotegerin (OPG) [85,171,205,206,207]. Ascites also contains exosomes which are able to transfer miRNAs, lipids, cytokines and growth factors, as well as OCSC marker molecules such as CD44 or EpCAM, able to pass signals between OCSCs and the TME from the primary tumor and implants [58,208,209].

#### 5.2.3. Peritoneal Implants—Mechanosensory Signals

One of the mechanisms of OCSCs stimulation in peritoneal implants is a response of cancer cells to mechanic stimuli and stress produced by peritoneal extension due to ascitic fluid. The activation of mechanosensory signals involves the YAP/ tafazzin protein (TAZ) signaling pathway, and accessory NF-κB, ERK, focal adhesion kinase (FAK), and Rho/Rho-associated protein kinase (Rho/ROCK) pathways. Mechanical stressors that influence the behavior of OCSCs in ovarian cancer include shear and compression produced by ascites, tension and compression caused by tumor growth against surrounding tissue, and stiffness resulting from ECM remodeling (desmoplastic reaction). Mechanosensory signals regulate EMT, change cancer cell morphology, enhance OCSC populations, increase CSC’s chemoresistance, increase angiogenesis, and regulate the interaction with ECM [179]. In detail, shear stress stimulates EMT, up-regulates chemo-resistance of OC cells, and enhances stemness via the stimulation of CD44, CD117 and OCT4 activity [210,211,212]. Tension and ECM stiffness activate the Rho/ROCK pathway and regulate EMT. Tension also influences VEGF-mediated angiogenesis in endothelial cells [179,213,214]. ECM stiffness stimulates the expression of CD133 stemness marker [195,215]. Compression may change the activity of genes regulating EMT and the activity of the Wnt/β-catenin pathway [216].

#### 5.2.4. Peritoneal Implants—Mesothelium

Cancer cells from spheroids via the secretion of TGF-β induce mesothelial epithelium to produce fibronectin which augments OC cells’ adhesion and proliferation in new places. Exosomes liberated from the tumor are able to transfer CD44 protein into mesothelial cells stimulating metalloproteinase-9 (MMP-9) expression, which supports cell homing and invasion [217]. Moreover, CD133 molecule promotes the mesothelial attachment of floating OC cells [218]. Mesothelium cells are also capable to release soluble factors (such as lysophosphatidic acid, protein K90 and VEGF) into ascites which enhance apoptotic resistance, drug resistance and poor prognosis in HGOC patients [219,220,221,222,223].

#### 5.2.5. Hematogenous Distant Metastases

Alternative to passive transcoelomic metastases is an active hematogenous route of metastases into the omentum described in the conjoined guest mouse model. In this model circulating in blood, OC cells metastasize firstly into the omentum through the vasculature and then disseminate into the peritoneal cavity. The human epidermal growth factor receptor-3 (ErbB3)/Neuregulin-1 signaling pathway was shown to play a key role in this mechanism [224]. A hematogenous way of metastases into distant parenchymal organs, such as liver, lungs, lymphatic nodes, and brain is dependent on circulating CTCs which possess characteristics of OCSCs showing positive markers of stemness (CD44v6, CD117, ALDH1A1, NANOG, OCT4) and EMT (N-cadherin, vimentin, SLUG) [63,225].

#### 5.2.6. Cellular Components of Metastatic Niche

**Figure 1:**
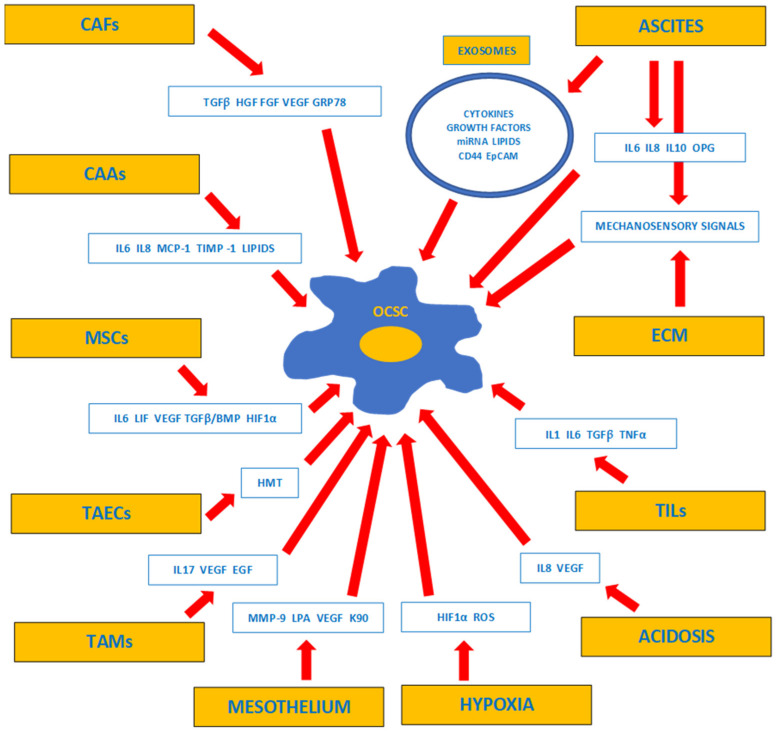
Secretory and mechanosensory signals identifying ovarian cancer-specific tumor microenvironment (TME) and ovarian cancer stem cells’ (OCSCs) niche existing inside peritoneal cavity. Ascites is a unique microenvironment for OCSCs, which contains interleukins IL-6, IL-8, IL-10, vasculo-endothelial growth factor (VEGF), osteoprotegerin (OPG), exosomes (which transfer miRNAs, lipids, cytokines, growth factors, and OCSCs markers CD44 or EpCAM) able to pass signals between TME and OCSCs. Mechanosensory signals from ascites comprise shear and compression stress. Tension, stiffness and desmoplastic reaction are other mechanic stressors resulting from extracellular matrix (ECM) remodeling. Cancer-associated fibroblasts (CAFs) secrete transforming growth factor-β TGF-β which stimulates epigenetic changes promoting epithelial-mesenchymal transition (EMT) and metastases. CAFs also secrete hepatocyte growth factor (HGF), glucose-regulated protein 78 (GRP78) which augment invasiveness and chemo-resistance, as well as fibroblast growth factor (FGF) and VEGF both stimulating angiogenesis and chemo-resistance. Adipocyte-derived IL6, IL8, monocyte chemoattractant protein-1 (MCP-1) and tissue inhibitor of metalloproteinase-1 (TIMP-1) recruit cancer cells into the surface of the omentum. Cancer-associated adipocytes (CAAs) feed also OCSCs with lipids. LIF and IL-6 secreted by mesenchymal stem cells (MSCs) promote OCSC’s function. Up-regulation of TGF-β/bone morphogenic protein (BMP), VEGF and HIF-1α contributes to angiogenesis and stimulates OCSCs phenotype. Secretion of IL-17, VEGF and epidermal growth factor (EGF) by tumor-associated macrophages (TAMs) promotes OCSCs phenotype, thus supporting peritoneal carcinomatosis and implant formation. Pro-inflammatory cytokines (transforming growth factor-β (TGF-β), tumor necrosis factor-α (TNF-α), IL-1, IL-6) produced by activated tumor-infiltrating lymphocytes (TILs) enhance EMT. Tumor-associated endothelial cells (TAECs) secrete enzyme histone-lysine N-methyltransferase (HMT) which increases OCSC’s stemness. Mesothelium cells release soluble factors (such as lysophosphatidic acid (LPA), protein K90 and VEGF) into ascites which stimulate tumor aggressiveness and chemo-resistance. Hypoxia and acidosis in tumor TME are the stimulators of EMT and OCSCs stemness via hypoxia-inducible factor-1α (HIF-1α), reactive oxygen species (ROS) and IL8, VEGF, respectively.

Once homed into a peritoneal or distant organ environment, OC cells start to cooperate with several cell populations to create a metastatic niche. Major populations of TME cells participating in this process are CAFs, CAAs, MSCs, TAMs, TILs, tumor-associated endothelial cells (TAECs), and pericytes. Figure 1 presents main secretory and mechanosensory signals identifying ovarian cancer-specific TME and OCSC niches existing inside the peritoneal cavity.

##### Cancer-Associated Fibroblasts—CAFs

CAFs are a population of fibroblasts or transformed MSCs that play an indispensable role in the TME by affecting the progression, dissemination and chemo-resistance of OCSCs. The functional activation of CAFs depends on inflammatory signals and hypoxia [203,226,227,228,229,230]. OC cells are able to convert omental fibroblasts into CAFs by the secretion of chemokine CCL5 and exosomes containing miRNAs [231]. Activated CAFs secrete TGF-β, which stimulates epigenetic changes promoting EMT and metastases and in a positive feedback loop stimulating CAFs themselves to stronger tumor-promoting phenotypes [232]. The strong pro-tumoral activity of CAFs could be also stimulated by dickopf-related protein-3 (DKK3) which enhances YAP/TAZ and Wnt/β-catenin signaling pathways. Increased stromal expression of DKK3 has been correlated with aggressive behavior of OC tumors [164]. The expression of metalloproteinases and environments rich in ROS also are able to activate CAFs [179]. In OC, CAFs stimulate cancer invasiveness and chemo-resistance through the secretion of hepatocyte growth factor (HGF), glucose-regulated protein 78 (GRP78), and the activation of hepatocyte growth factor receptor HGFR (also known as cMet)/PI3K/AKT signaling pathway [233,234]. The activation of insulin growth factor-1 receptor (IGF-1R)/AKT signaling by CAFs in response to chemotherapeutic drugs induces chemoresistance and stemness of OC cells by an increase in NANOG, OCT4 and SOX2 [234]. Moreover, CAFs stimulate OCSCs via fibroblast growth factor (FGF) to autocrine VEGF-A secretion. VEGF-A through VEGF-R2 receptor activates proto-oncogene tyrosine-protein kinase Src-dependent up-regulation of stem cell factor B-cell specific Moloney murine leukemia virus integration site-1 (Bmi1), thus augmenting the stemness of OCSCs [235]. The secretion of VEGF by CAFs also affects tumor endothelial cells, thus influencing angiogenesis and chemo-resistance, whereas the secretion of MMPs, ECM components, and enzymes helps to remodel the TME according to the tumor demands [236,237]. CAFs also change immune balance inside the tumor by the suppression of cytotoxic TILs and the promotion of pro-inflammatory signals [238,239].

##### Cancer-Associated Adipocytes (CAAs) and Lipid Metabolism

Adipose tissue plays an extraordinary role in the growth of ovarian cancer (omentum, mesentery, large bowel appendices and small fatty foci inside parietal and diaphragmatic peritoneum). Adipose tissue stores lipids but has been also recognized as a regulatory and secretory organ capable to produce adipokines, metabolic signals, growth factors, hormones and immune mediators. Apart from adipocytes, the stromal components of adipose tissue have a significant contribution to this function [240]. Omental implants are a classic example of OCSC niches, in which adipocytes play an important role in nesting and the proliferation of OCSCs [219]. IL-6, IL-8, monocyte chemoattractant protein-1 (MCP-1) and tissue inhibitor of metalloproteinase-1 (TIMP-1) are responsible for the tropism between CSCs and adipocytes, and help to recruit cancer cells into the surface of the omentum. The interaction between IL-8 from adipocytes and C-X-C chemokine receptor type-1 (CXCR1) on cancer cells activates the p38MAPK/STAT3 phosphorylation pathway initiating metastasis [241,242]. Inflammatory signals from OC cells mobilize omental neutrophils and stimulate them to create chromatin webs called “neutrophil cellular traps” (NETs) in early-stage OC patients. NETs capture floating cancer cells and help to initiate omental implants [243]. OC cells rely on lipogenesis to survive stress in the TME, especially in hypoxic conditions. Adipocyte-derived lipid transfer into OC cells depends on fatty acid binding protein-4 (FABP4), which is up-regulated in metastatic compared to primary tumors. High levels of fatty acid desaturation and oxidation in FABP4-expressing tumors correlate with poor survival in patients [244]. Desaturation and oxidation of fatty acids are responsible for sustaining cancer cell membrane integrity, inter-cellular signaling, and energy production [245,246]. ALDH+CD133+ OCSCs in spheroids indicate increased levels of unsaturated lipids compared to non-CSCs, and the inhibition of desaturation eliminates OCSCs in vitro and in vivo [247,248]. The survival of OCSCs depends critically on the proper function of stearoyl-CoA desaturase-1 (SCD1) regulated by the NF-κB pathway, and the elimination of SCD1 activity also eliminates OCSCs. Intracellular monounsaturated fatty acids provide appropriate energy input for EMT transition [249]. Fatty acid synthase (FASN) is an important enzyme engaged in lipogenesis. FASN expression correlates with OC tumor clinical staging and histological malignancy, and patients showing increased FASN expression have a worse survival rate and platinum resistance [250,251].

##### Mesenchymal Stem Cells (MSCs)

Mesenchymal stem cells are recruited into the tumor stroma from different locations including bone marrow, adipose tissue, and endometrium, and act as promotors of OCSCs or differentiate into other active components of OCSC niches such as CAFs. Through interactions with OCSCs, they stimulate OC proliferative potential, stemness, platinum-resistance and neovascularization [252,253,254,255,256]. Interactions with MSCs up-regulate the PI3K/AKT pathway and multi-drug resistance (MDR) proteins in OCSCs, resulting in resistance to paclitaxel and carboplatin [257,258,259,260,261]. LIF and IL-6 secreted by MSCs promote OCSCs’ function by STAT3 signaling [262]. Enrichment of the tumor OCSC population by MSCs is mediated by the up-regulation of TGF-β/ bone morphogenic protein (BMP) family members [263]. The secretion of VEGF and the overexpression of HIF-1α contributes to angiogenesis, and also up-regulates OCSC phenotypes [264]. However, depending on environmental signals, the epigenetic regulation MSCs could have also a tumor-restrictive capability [265,266].

##### Tumor-Associated Macrophages—TAMs

TAMs have a multifunctional influence on cancer cells. They are engaged in cancer-associated inflammation, immune escape, angiogenesis and invasion, and finally stemness. All those actions are attributed to the TAM’s immunosuppressive M2 phenotype. Hypoxic OC tumors could program M2 polarization of TAMs through exosomal miRNAs miR-222-3p and miR-940 and the activation of the suppressor cytokine signaling-3 (SOCS3)/STAT3 pathway [267,268]. The presence of cytokines IL-4, IL-10, and IL-13 secreted by OC cells and MSCs in the tumor niche is another signal for M2 polarization [269]. LIF and IL-6 from ascites also promote monocyte conversion into TAMs [270]. Increased activity of JNK and NF-ĸB pathways facilitate cancer cell invasion by TAMs [271]. The secretion of pro-inflammatory IL-17 by TAMs promotes OCSC phenotypes through NF-ĸB/p38MAPK signaling [71]. VEGF, produced by TAMs, is responsible for peritoneal carcinomatosis [242]. Intraperitoneal TAMs promote OC spheroid and implant formation through the secretion of the epithelial growth factor (EGF) [271,272]. M2-polarized TAMs, together with MDSC suppressor cells, support tumor growth deviating host immunity into a pro-tolerance state characterized by the inhibition of natural killer (NK) and cytotoxic T cells activity, and the activation of Tregs [273,274,275,276,277]. Pro-inflammatory cytokines (TGF-β, TNF-α, IL-1, IL-6) produced by activated TILs in a hypoxic tumor environment enhance EMT through the NF-ĸB/STAT3 pathway [253].

##### Tumor-Associated Endothelial Cells (TAECs) and Pericytes

Endothelial cells which line pathological tumor vessels have an improper morphology and molecular profile and are called TAECs. They are responsible for increased vascular permeability of the intra tumor vasculature and support cancer cells in metastasizing. They also augment stemness by helping to overcome anoikis and to develop resistance to drugs in OC cells. A significantly up-regulated enhancer of zeste homolog-2 gene (*EZH2*) coding a histone-lysine N-methyltransferase was described in TAECs in OC. This enzyme is an epigenetic stimulator of OCSC stemness, cancer invasiveness, metastatic potential and chemo-resistance [278,279,280,281]. Another population of vascular cells constituting a component of TME are pericytes, which physiologically provide physical support to endothelial cells. OC pericytes contribute to neoangiogenesis, but also the expansion of OCSC populations [282].

#### 5.2.7. Hypoxic Environment

CSCs show unique adaptation to variable levels of tissue oxygenation inside tumors, and are capable of using both aerobic glycolysis and oxidative phosphorylation (OXPHOS) [15]. In normoxic areas of the tumor, CSCs prefer OXPHOS; although, in hypoxic conditions CSCs can switch to aerobic glycolysis. However, even in a hypoxic environment, cancer cells in most cases simultaneously use OXPHOS and glycolytic metabolic pathways. CAFs support CSCs’ metabolic reprogramming and help to remove lactates in the so-called “reverse Warburg effect” [15,196]. The key glycolytic enzyme converting glucose into glucose-6-phosphate is hexokinase-2 (HK2). Its high expression has been observed in serous HGOC and been correlated to chemo-resistance [283,284]. One of the mechanisms which can suppress OXPHOS in mitochondria is the inactivation of pyruvate dehydrogenase by 3-phosphoinositide-dependent protein kinase-1 (PDK1). The up-regulation of PDK1 was also noticed in HGOC tumors and has been linked to chemo-resistance and unfavorable outcomes [285,286,287]. CD44+CD117+ OCSCs found in ascites of OC patients showed enhanced glucose uptake and increased OXPHOS function with higher ROS production [102,288]. Mitochondrial-associated granulocyte-macrophage colony-stimulating factor-signaling molecule (MAGMAS) regulates the ATPase activity of the inner membrane protein import motor in mitochondria. The loss of MAGMAS activity impairs oxidative phosphorylation followed by the increased accumulation of ROS and cell cycle arrest, whereas overactivity protects cellular viability. The overexpression of MAGMAS was noticed in HGSOC and was even higher after therapy with paclitaxel indicating its importance in chemo-resistance and stemness [289,290,291,292]. Hypoxia inside OC tumors triggers a high expression of the HIF-1α transcription factor. HIF-1α enhances the activation of EMT and stemness activators such as Wnt/β-catenin, Hedgehog, NOTCH signaling pathways, and CSCs markers such as CD133, NANOG, and SOX2 [197,198]. Acidosis is another hallmark of the TME and CSC niche. Acidic conditions are a direct consequence of glycolytic activity and the conversion of pyruvate into lactate, as well as the production of carbon dioxide during mitochondrial respiration. Acidosis stimulates the efficacy of the OXPHOS mechanism. It also regulates drug resistance, cancer cells dormancy and autophagy [199]. Acidosis increases the expression of OCT4 and NANOG in CSCs, as well as the secretion of VEGF and IL8 in the CSCs niche [199,293,294,295,296]. Acidification of the TME inhibits the function of T cell effectors against cancer and stimulates the polarization of T cells into pro-tolerant Tregs [297].

## 6. Genetic and Epigenetic Regulation of OCSCs

Defective genes (i.e., *CTNNB1, PTC, SMO, NOTCH, k-Ras, b-Raf,* and *MEK*) disturb the function of Wnt/β-catenin, Hedgehog, NOTCH, RAS/MEK, or PI3K signaling pathways in OCSCs [298]. Similar consequences include a loss of expression of *BRCA* genes followed by the activation of the PI3K-signaling pathway [299]. In OC, dysregulated *BRCA* and *TP53* gene expression is accompanied by the deregulation of genes responsible for the function of the centrosome, cell membrane receptors, and cell cycle, such as *NAB1, PROS1, GREB1, KLF9* [276,298]. Chromosome instability resulting from the influence of TME in the peritoneal cavity and ascites on the cancer genome has been noticed in HGOC. Germline mutations of DNA double-strand break repair system genes (*RAD51C, RAD51D, BRIPI, BARDI*) and mismatch repair genes (*MSH2, MLH1, MSH6*) have also been observed in OC [300,301].

### 6.1. Non-Coding RNA

The epigenetic change of gene expression is one of the most important factors responsible for CSCs’ plasticity. Signals originate directly from the TME and CSC niche, or are delivered to the CSCs via exosomes. Small non-coding regulatory micro RNAs (miRNAs) are capable to change the expression of target genes and can function as both stimulators and suppressors of CSC stemness, self-renewal, proliferation, migration, and chemo-resistance. The function of CSCs could be also regulated by long non-coding RNAs (lncRNAs) defined as RNA transcripts exceeding 200 nucleotides but not translated to proteins. They participate in the regulation of gene transcription, as well as in post-translational and epigenetic regulation. Cultured OC cells possess an aberrant expression of miR-200 family miRNAs, mainly miR-204, miR-206, miR-100, miR-200a, miR-200b, miR-200c, miR-141, miR-429 and miR-223; however, CD133+ OCSCs are exclusively characterized by a decreased level of miR-200a. This miRNA increases the migratory potential of OCSCs and stimulates EMT. Down-regulation of miR-200a is required for the maintenance of OCSCs [70,302]. Another family of miRNAs deregulated in OC is a Let-7 family consisting of Let-7a to Let-7l members and miR-98 and miR-202. Let-7 miRNAs act through the regulation of oncogenes such as *MYC*, *K-Ras* or high-mobility group AT-hook-2 (*HMGA-2*). The deregulation of Let-7 sustains a stem phenotype of OCSCs. The interplay between Let-7 and its inhibitor Lin-28 homolog A RNA-binding protein (LIN28) regulates the biology of OCSCs. LIN28 expression in ovarian cancer specimens correlates with and maintains ALDH1 expression, while Let-7 over-expression reduces ALDH1+ OCSCs [303]. Another two miRNAs deregulated specifically in OC are miR-214 and miR-199a. MiR-214 targets *PTEN* leading to PTEN down-regulation and the activation of the AKT pathway. The up-regulation of miR-214 enhances cell survival and chemo-resistance. MiR-214 affects OCSCs by targeting the p53/NANOG pathway and the over-expression of miR-214 inhibits p53, thus stimulating the stemness of OC cells [304]. MiR-199a targets inhibitors of nuclear factor kappa-B kinase subunit-β (IKK-β) and regulates the NF-κB-dependent inflammatory environment in OC [305]. The expression of miR-25-3p and miR-15a-5p regulates the proliferation of OC, and the inhibition of both miRNAs combined with docetaxel results in a decrease in cell divisions and an activation of apoptosis [306]. The decreased expression of miR-136 is associated with primary platinum-resistance; however, miR-136 over-expression inhibits the activity of OCSCs and restores chemo-sensitivity [307,308]. Lower levels of miR-146a in primary tumor tissue samples were correlated with a shorter progression-free survival and platinum-resistance of peritoneal metastases [309]. Low expression of miR-15a and miR-16 in HGOC correlates with the up-regulation of Bmi1 and the activation of stemness in OC cells [310]. Another miR-506, by targeting SLUG, a transcriptional negative regulator of E-cadherin, provides E-cadherin higher expression and the prevention of TGF-β-induced EMT. OC samples showing miR-506 overexpression were correlated with beneficial prognosis for patients [311]. Ovarian cancer cells are able to convert TME fibroblasts into active CAFs by the up-regulation of miR-155 and the down-regulation of miR-31 and miR214, followed by changes in chemokine secretion (CCL-5, CXCL-10) which reciprocally enhance tumor growth [231]. Increased CXCL-14 expression by CAFs also produces the up-regulation of lncRNA LINC00092 in OC cells which facilitates glycolytic activity of the tumor [312]. Omental CAFs and CAAs down-regulate OC pro-apoptotic pathways and augment chemo-resistance of peritoneal implants by the transfer of miR-21-containing exosomes [313]. Similarly, MSCs can positively influence OC growth by the secretion of exosomes containing miR-21, miR-221 and miR-92a [314]. Tumor-associated endothelial cells, in response to VEGF, overexpress EZH2 which stimulates tumor angiogenesis by the inhibition of vasohibin-1. The expression of EXH2 in OC cells is regulated by miR-298 and homeobox HOX transcript antisense lncRNA HOTAIR [315,316]. TAM-originating exosomes containing miR-146b-5p activate the TNF receptor-associated factor protein-6 (TRAF6)/NF-ĸB/MMP2 pathway and suppress endothelial cell migration inside tumors [317]. TAMs activated by the TNF-related inducer of apoptosis (TWEAK) expressed by activated immune cells secrete exosomes containing miR-7, increasing the suppression of OC cells’ metastatic activity by the down-regulation of the EGFR/AKT/ERK1/2 pathway [317,318]. Many more miRNAs and lncRNAs engaged in OC regulation have been recognized; however, the targets are not known for all of them. Part of miRNAs cause the loss of tumor suppressor function targeting *CDCP1, PLGL2* genes (miR-654-5p), *APC2* gene (miR-939), or *SFRP1* gene (miR-1180, miR-1207) [319,320,321,322]. Another group acts through the inhibition of Wnt-signaling (miR-15b, miR-16, miR-200c, miR-219-5p) [254,323,324], while others activate Wnt-signaling (miR-29), followed by enhanced tumor growth [325,326]. Most of the lncRNAs studied in OC also act to either activate (HOXB-cluster antisense RNA-3—HOXB-AS3; associated with poor prognosis of hepatocellular carcinoma—AWPPH; metastasis-associated lung adenocarcinoma-1—MALAT1; colon cancer-associated transcript-2—CCAT2; small nucleolar RNA host gene-20—SNHG20) or deactivate (HOXD-cluster antisense RNA-1—HOXD-AS1) the Wnt-signaling pathway [327,328,329,330,331,332]. Profiling studies have revealed that the metastasizing activity of HGOC cells is regulated by the overexpression of seven lncRNAs (FLJ39739, GAS5, H19, LOC100499466, MALAT1, NEAT1, TUG1) and the low expression of four lncRNAs (CASC2, DLEU2, HCG18, LOC100133669) [333]. The most recent studies show a clear connection between some lncRNAs and OCSC function. Transfection of OC cell cultures with the WDFY3-AS2 lncRNA resulted in the increased expression of SOX2, OCT4 and NANOG, as well as in enrichment of CD44+CD166+ sphere-forming cells in the culture. The transfection of CD133+ OCSCs with LINC00115 lncRNA promoted stemness and inhibited apoptosis of OCSCs by up-regulating SOX9 and the Wnt/β-catenin pathway [334]. In SKOV3 and OVCAR3 cell cultures, the lncRNA T-cell factor-7 (lnc-TCF7) was found to be up-regulated, followed with decreased apoptosis and increased CD44, CD133 expression and CD44+CD133+ cell proportion with concomitant spheres formation efficiency and drug resistance against cisplatin [335]. In HGOC cell lines (OVCAR3, CAOV3, OVCAR5, COV362, Kuramochi), lncRNA HOTAIR was generally overexpressed, but its expression was especially up-regulated in ALDH+ OCSCs and associated with increased spheroid formation and colony forming ability [336].

### 6.2. Defective DNA and Histone Methylation

Another mechanism of epigenetic regulation in CSCs is the hypermethylation of CpG islands of promoter genes, histones, and non-histone proteins, which is associated with either the activation or silencing of the regulated gene. The hypermethylation of DNA in cancer may contribute to the formation of CSCs [337,338]. Methylation is dependent on the function of DNA methyltransferases (DNMTs) which were found to be up-regulated in OC cells [339]. In ovarian cancer, CpG islands of many tumor-suppressor genes are hypermethylated, including *PTEN, SLIT2, MLH1, RASSF1A,* and *BRCA1*, even in the early stages of the disease [340,341,342]. Hypermethylation results in DNA-repair loss (*BRCA1*) and cell cycle control disturbances (*PTEN, RASSF1A*). In response to chemotherapy in OC patients, the hypermethylation of genes responsible for cell resistance to apoptosis has been observed (*DAPK, LOT1, PAR4*) [289]. The methylation of H3K27 histone causes the silencing of miR-200c and miR-205 expression, thus activating EMT transition and CSC phenotypes [343]. Histone methylation is also responsible for the increased expression ABC of transmembrane transporters responsible for chemo-resistance of CSCs [326]. The dysregulated function of histone acetyltransferases (HAT) and deacetylases (HDAC) are also connected to cancer progression, and the over-activity of HDAC1 and HDAC7 enzymes promotes stemness in CSCs in ovarian cancer [344,345,346]. Hypomethylation is the next epigenetic regulator of OC behavior. DNA hypomethylation takes place in repetitive parts of the genome, such as the interspersed retrotransposon LINE-1. Hypomethylation of LINE-1 has been found both in precursor lesions for HGOC in the tubes (STIC), and in advanced HGOC tumors, and is correlated with reduced survival [347].

## 7. Autophagy of OCSCs

Autophagy is a process of a self-digestion of proteins, lipids, and damaged cellular organelles inside phagosomes followed by the recycling of digestion products. During stressful conditions produced by hypoxia, starvation or toxic drug autophagy is a way to cell survival. The removal of dysfunctional mitochondria from the cell is called mitophagy [348,349]. Physiologically, autophagy protects against tumor initiation in the mutagenic environment, but after the cells have been transformed into cancer it helps to maintain tumor growth in a hostile environment. Autophagy protects cancer cells from pro-apoptotic stimuli and genome instability. It modifies anti-tumor immune response, regulates EMT, CSC dormancy, and chemo-resistance [350,351,352,353]. The abundance of nutrients and growth factors activate PI3K/AKT and the mechanistic (mammalian) target of rapamycin (mTOR) pathways, which inhibit autophagy. The absence of nutrients or hypoxia act contrarily and through the MAPK pathway autophagy is activated [354]. Autophagy changes the cell secretome system and increases pro-inflammatory cytokine and chemokine release by both cancer cells and TME components, thus influencing OCSCs’ functions [355]. Autophagy also plays an important role in the dormancy of cancer (most probably DTCs and CSCs cells). The down-regulation of tumor suppressor gene DIRAS3 in the majority of ovarian tumors reverses dormant cells into proliferative status. DIRA3 induces autophagy in dormant cells that enhances the survival of nutrient-deprived cells that remain after conventional chemotherapy [356,357].

## 8. OCSCs and Escape from the Host Immune Surveillance

### 8.1. General Considerations for Immune Escape of Cancer Stem Cells

Cancer stem cells have created mechanisms which enable them to escape from the host immune surveillance. In several human cancers, tumor-initiating cells equated to CSCs are able to reduce both the expression of human leukocyte antigens (HLA)—A, B and C—and antigen-processing machinery (APM) molecules, thus avoiding recognition by CD8+ cytotoxic T cells [358,359,360,361,362]. Cancer stem cells are also able to escape NK cell-mediated killing by the down-regulation of activating natural killer group 2D (NKG2D) ligands [358,360,363]. Additionally, CSCs express low levels or no ligands for the NK cell activator receptors NKp44, NKp30, NKp46 and CD16 [364,365,366]. Moreover, the ligation of immune checkpoint molecules (as programmed death-ligand-1—PD-L1; cytotoxic T-lymphocyte antigen-4-CTLA-4; B7 homolog 3—B7-H3 known as CD276; B7 homolog 4—B7-H4 also known as V-set domain containing T cell activation inhibitor-1—VTCN1) expressed on the surface of CSCs to their respective receptors on T lymphocytes results in a decrease in T cell proliferative and secretory abilities followed by the inhibition of IFN- γ-mediated anti-tumor activity and T cell apoptosis [367]. The molecule CD47 is the transmembrane protein that, through binding to the signal regulatory protein alpha (SIRPα) receptor on macrophages and dendritic cells (DCs), disables their phagocytic activity [368]. The up-regulation of the “don’t eat me” signal, mediated by the CD47 molecule in CSCs, is another mechanism providing the immune escape of CSCs, and it has been shown in several cancers that the blockade of the CD47 signal enables macrophage-mediated phagocytosis of CSCs [369,370,371]. The CSCS are also capable of down-regulation of the toll-like receptor 4 (TLR4)-mediated activity, thus escaping the innate immune response [372]. CSCs also convert the immature DCs into TGF-β-secreted cells, which support the expansion of regulatory T cells (Tregs) in lymphoid organs [373]. The CSCs can switch between a dormant and proliferative state, and during dormancy they are further resistant to host immune responses using several mechanisms, including the down-regulation of HLA antigens, and UL16-binding protein (ULBP) ligands which helps CSCs to evade T cell-mediated and NK cell-mediated cytotoxicity, respectively [374,375]. Dormant CSCs also evade T cell- and NK cell-mediated apoptosis through the genetic inactivation of the onco-suppressor caspase 8 (CASP8), inactivation of the surface death receptor Fas (FAS or CD95), and deregulation of the suppressor of cytokine signaling 1 (SOCS1) cascade [376,377]. Chronic stimulation of FAS in cancer cells was found to be connected to the activation of the stemness markers in several tumors [378].

The immune privileged status of CSCs is not dependent solely on the properties of the CSCs themselves, but depends strongly on the tumor micro-environment which, in the context of immune reactions, is called the tumor immune microenvironment (TIME). The major members of the CSCs TIME are Tregs, M2-differentiated TAMs, MDSCs and N2-differentiated tumor associated neutrophils (TANs). They all cooperate with CSCs to create an immunosuppressive environment through the secretion of several pro-tolerant cytokines such as IL-10, TGF-β, and prostaglandins, inhibiting the secretion of IL-12 by DCs, and thus blocking the effector cytotoxic Th1 response, as well as inducing a pro-angiogenic environment [379,380,381].

Although it is believed that the above-described phenomena are generally true, there are conflicting results which make the panorama of CSCs immune escape more complex. First of all, despite the fact that CSCs present HLA antigens defectively, they are still able to express tumor-associated antigens (TAAs) on their surface, including carcinoembryonic antigen (CEA), survivin, mucin 1 (MUC1) and cancer/testis antigen 1B (NY-ESO1) [382,383,384,385], which could serve as potential targets both for host immune reactions and for immunotherapeutic procedures. Secondly, the low expression of some HLA antigens on CSCs could be controverted by the fact that CD24+/CD44+/CD133+/ALDH+^bright^ CSCs were positive for MHC class I polypeptide-related sequence A (MICA), MICB, FAS and NK activator ligands, as compared to non-CSCs [381]. Animal studies on a murine model demonstrated that isolated murine cancer stem cell antigens (SCA)-1+ ID8 and CD133+ HM-1 CSCs were susceptible to phagocytosis and CD8+ T cell-mediated immunity [386]. The behavior of CSCs is the result not only of the CSC genome, but also of the signals originating from the TIME; the tumor advancement and even the tumor type (i.e., CSCs isolated from colorectal cancer expressed HLA-I molecules [387]), might explain the observed discrepancy of the results.

### 8.2. Immune Escape of OCSCs

The tumor microenvironment down-regulates MHC expression by OC cells [388]. Ovarian cancer has shown up-regulated CD47 expression as connected to worse OS and PFS in OC patients. Correlation was found between CD47 expression level and immune infiltration inside the tumor [389]. In a murine model, CD47 low-expression SCA-1+ ID8 ovarian CSCs were prone for rejection; however, the restoring of CD47 expression on SCA-1+ ID8 CSCs delayed their immune-mediated elimination. SCA-1+ ID8 ovarian CSCs also showed rapid growth by mixing with non-stem cancer cells, suggesting that OCSCs could be protected from immune attack by surrounding non-stem tumor cells residing inside the tumor niche [386]. It was also shown that OC typically expresses NKG2D ligands, which strongly correlate with negative disease outcomes [390,391,392,393]. The NKG2D ligands could be expressed either in soluble form in the extracellular space of the tumor, or on the tumor cell surface. In the first case, they function as decoy receptors binding the NKG2D molecules on antitumor effector γδ T cells, NK cells, and cytotoxic CD8+ T cells [394,395]. In the second case, the presence of NKG2D ligands in OC cells can trigger the self-stimulation of cancer cells by NKG2D molecules leading to the formation of self-renewal OCSCs and effective tumor generation [394]. One of the pathways which plays a key role in tumor immune escape is the interaction between tumor PD-L1 ligand and the corresponding T cell PD-1 receptor. The up-regulation of PD-L1 on the OCSCs forces the shift in T effector/Tregs balance towards tumor tolerance enhancing its proliferation. Increased tumor expression of the PD-L1 ligand correlates to bad prognosis in OC patients [396].

OCSC niches are very specific because of the inflammatory and immunosuppressive milieu of the peritoneal cavity, where OC implants show exceptional tropism towards fatty tissue (omentum, colon appendices) and highly vascularized peritoneal immune collections called milky spots, which are followed by the production and accumulation of ascites [397,398]. Ascites is rich in immunosuppressive cytokines and contains increased numbers of immune tolerant PD-1 and CTLA-4 expressing T cells [399,400]. This unique environment also plays an important role in the creation of specific TIME where several immune cell types, including TAMs, TANs, DCs, Tregs, and MDSCs cells support tumor progression, chemoresistance, and immune evasion [401]. Neutrophils establish the premetastatic omental niche forming the neutrophil extracellular traps (NETs), which have been found in OC-bearing mice even before the emergence of omental metastases in human patients with early-stage OC [243]. The formation of omental NETs is regulated by the secretion of ROS, IL-8, granulocyte colony stimulating factor G-CSF and growth-regulated oncogene (GRO) α by early OC tumors, and is responsible not only for the establishment of cancer implants but also for the activation of dormant and cancer stem cells [243,402]. Hypoxia present inside the peritoneal environment and especially inside growing tumors activates hypoxia-dependent signaling, where HIF-1α plays an important role between others by the promotion of MDSCs, which produce TGF-β, IL-6, and IL-8, contributing to immunosuppression in advanced OC [403]. Cytokine TGF-β subsequently induces the population of pro-angiogenic N2-polarized TANs supporting tumor growth and neo-vascularization [379]. MDSCs also induce the expression of microRNA101 in ovarian cancer cells, which subsequently increases their stemness and tumorigenic potential [404]. Hypoxia also attracts macrophages supporting immune tolerance against tumor cells, including OCSCs [405]. OCSCs are capable of releasing cycloxygenase2 and chemokine (C-C motif) ligand 2 (CCL2), which polarize TAMs into M2 activity which, in turn, contributes to the maintenance of OCSC stemness [406]. Regulatory T cells (Tregs) are recruited by ovarian cancer cells via the CCL22 and TGF-β pathways [407]. Hypoxic conditions up-regulate chemokine (C-C motif) ligand 28 (CCL28 also known as mucosae-associated epithelial chemokine—MEC) which attracts Tregs; this, in turn, down-regulates the effector T cell responses [408]. Tregs are additionally recruited towards TIME by chemokine (C-C motif) ligand 5 (CCL5)—C-C chemokine receptor type 5 (CCR5) interactions—dependent on the high CCL5 expression of OCSCs, as well as increased expression of indoleamine 2,3-dioxygenase 1 (IDO1) and C-X-C motif chemokine ligand 2 (CXCL2) on OCSCs [409]. Tregs cultured in conditioned medium from OCSCs exhibited increased IL-10 and MMP-9 expression which enhanced the tumor invasion [409]. Moreover, prolonged exposure of DCs to hypoxia resulted in decreases in stimulatory cytokine IL-12 secretion and finally in DC cell death [410,411]. The described nets of interactions between OCSCs and TIME provide a strong support for OCSCs’ immune escape, and are among the major obstacles in successful anti-tumor treatment.

## 9. Anti-OCSC Therapy

Cancer stem cells constitute a very attractive target for therapy. The effective elimination of this cell population would probably improve the treatment of advanced cancer and protect against recurrent lethal disease. Therefore, many different approaches to CSC-targeted management have been proposed and tested in vitro, in experimental settings and in clinical trials (Table 2). CSCs are under extensive investigation in practically all known types of cancer. The most popular and tested targets for anti-CSC therapy are signaling pathways regulating the origin and function of CSCs, the surface and intracellular markers of CSCs, drugs changing the epigenetic regulation of CSCs’ function, and CSCs’ metabolism. Among them, there are inhibitors of Wnt (Ipafricept), Hedgehog (Vismodegib, Sonidegib), NOTCH (enoticumab, demcizumab, navicixizumab), MAPK (Salinomycin), and other signaling pathways (Metformin). Another group are drugs targeting CSC markers (Imatinib mesylate), epigenetic regulation by DNA-(cytosine-5)-methyltransferase-1 (DNMT1) (Decitabine, Guadecitabine, Azacitidine) or by histone deacetylase (HDAC) (Vorinostat, Belinostat, Etinostat). There are also some natural compounds being tested, such as curcumin, which has indicated anti-cancer activity in in vitro and animal studies. Another approach is to combine drugs or toxic agents with nanoparticles capable to transport them precisely into the tumor (examples are glucose-coated gold particles, paclitaxel albumin-bound nanoparticles, doxorubicin or mangostin encapsulated poly D/L lactide-co-glicolide acid—PLGA) [412]. Immunotherapy directed against tumor antigens with the use of chimeric antigen receptor T cells (CAR-T lymphocytes) has also been tested in many malignancies [413]. However, the immunosuppressive environment of solid tumors represents a barrier to this therapy’s success, due to low antigen expression on tumor cells [414]. A combination of several methods, for example, CAR-T cells and oncolytic viruses (Ovs), can allow the targeting of CSCs and the surrounding cancer niche. An OV-based strategy to overcome the mechanism of CAR-T cell evasion is to encode CAR-targeted TAAs in OVs to increase TAA expression more homogeneously across the tumor [415].

In the next paragraphs, we focus on the most advanced clinical trials targeting OCSCs. They concentrate on inhibitors of cancer stem cells’ signaling pathways, DNA (cytosine-5)-methyltransferase 1 (DNMT1), and histone deacetylase (HDAC) inhibitors.

### 9.1. Wnt-Signaling Inhibitor

Ipafricept (OMP54F28) is a fusion protein composed of the domain of the frizzled 8 receptor and the human immunoglobulin Fc domain which competes with the membrane-bound frizzled 8 receptor for Wnt proteins, thus inhibiting the Wnt-signaling pathway. Ipafricept has been tested in an NCT02050178 Ia/Ib phase clinical trial designed to determine the maximum tolerated dose and treatment regimen in combination with platinum and taxane standard chemotherapy. The number of 37 patients with platinum-sensitive recurrent OC were treated with adverse effects as followed: fatigue (40%, nausea (35%), diarrhea (22%), low appetite (22%), dysgeusia (19%), and vomiting (16%). In 22% patients, grade ≥ 3 treatment-related adverse effects were reported, mainly neutropenia. The tested protocol was considered as being well tolerated; however, neutropenia could be a serious limiting factor in treatment efficacy [462].

### 9.2. Hedgehog Signaling Inhibitors

Vismodegib (GDC-0449) is the smoothened (SMO) antagonist inhibiting the Hedgehog-signaling pathway which has been FDA-approved for the treatment of basal cell cancers. In an NCT00739661 II phase clinical trial, 104 patients with recurrent EOC, peritoneal, or fallopian tube cancer after the second or third complete remission were randomized to vismodegib (n = 52) or placebo (n = 52), maintaining monotherapy for 14 weeks. The median PFS in the drug and placebo arm was 7.5 months versus 5.8 months, respectively, and did not reach the expected value, probably due to the low expression of Hedgehog ligand in the patients’ archive tissue samples. The frequency of grade 3/4 adverse effects was 23%, and the most common side effects comprised dysgeusia, muscle contractions and alopecia [418]. Another Hedgehog-signaling pathway inhibitor, sonidegib (LDE225), was tested in escalating dose levels combined with paclitaxel in an NCT02195973 II phase clinical trial in 18 patients with advanced solid tumors including ovarian cancer. In OC patients, treatment resulted in a partial response or the stabilization of the disease during the therapy with maximal doses of sonidegib. In 30% of patients the maximum dose had to be reduced due to the toxicity. The clinical response was surprisingly not correlated to the immunohistochemical staining of the archival tissue samples for Hh-signaling biomarkers (SMO, Patched, SHH, GLI1) [463].

### 9.3. NOTCH Signaling Inhibitor

Demcizumab (OMP-21M18) is an anty-delta-like ligand-4 (DLL4) IgG2 humanized monoclonal antibody inhibiting the NOTCH signaling pathway. The open-label SIERRA Ib phase clinical study was performed in order to investigate the efficacy and tolerance of demcizumab in combination with weekly paclitaxel in platinum-resistant ovarian, peritoneal, and fallopian tube cancer in 19 pretreated patients with ≤4 cycles of chemotherapy. Overall response rate (ORR) was 21% and the most common side effects observed were diarrhea (68%), fatigue (58%), peripheral edema (53%), and nausea (53%). In three patients pulmonary hypertension was observed. The combination of demcizumab with paclitaxel has, according to authors, a manageable toxicity profile and has shown moderate activity against heavily pretreated ovarian tumors [424].

### 9.4. PI3K/mTOR/ERK/STAT3 Signaling Modulator

Metformin is the biguanide-derivative which functions as the modulator of cellular metabolism mainly through augmenting the tissue response for both endogenous and exogenous insulin. In cancer patients, the probable mechanism of function is based on the stimulation of adenosine monophosphate-activated protein kinase (AMPK) followed by the inhibition of PI3K, mTOR and ERK/STAT3 signaling pathways, the deprivation of energy support for cancer cells and their apoptosis [464]. Metformin has been tested as an OCSC-targeting drug in a II phase NCT01579812 clinical trial in 38 patients with FIGO IIC-IV EOC. Patients were treated according to the following protocols: 1/neoadjuvant metformin, debulking surgery, and adjuvant standard chemotherapy, plus metformin; or 2/neoadjuvant chemotherapy and metformin, interval debulking surgery, and adjuvant standard chemotherapy plus metformin. Median PFS was 18 months, and median OS was 57.9 months. Metformin-attributed side effects were observed, mainly diarrhea (18%), nausea (16%), elevated liver enzymes (21%), abdominal pain (11%), and vomiting (8%). Grade 3/4 adverse effects were noticed in two patients (diarrhea and skin rash). Pathological samples of metformin-treated tumors indicated an over two-fold decrease in ALDH+CD133+ OCSCs and an increase in sensitivity to cisplatin ex vivo. Moreover, metformin altered the methylation signature in cancer-associated MSCs, which reversed the MSC-driven chemoresistance in vitro [465].

### 9.5. Protein Kinase KIT (CD117) Inhibitor

Imatinib mesylate (Gleevec^®®^) inhibits the tyrosine protein kinase KIT (CD117) and the platelet-derived growth factor (PDGF)-regulated pathway, followed by programmed cell death. The efficacy and tolerability of imatinib mesylate in 24 heavily pretreated (median: four courses of chemotherapy) patients with recurrent metastatic ovarian and primary peritoneal cancer was tested during NCT00510653 II phase clinical trial. The stabilization of the disease during a median follow-up of 6.6 months was documented in 33% of patients, however, no partial or complete responses were noticed. Tissue expression of c-Kit and PDGF targets was shown in 50% and 94% of samples, respectively, but without relationship to the best response rate. Adverse events, mainly benign (fatigue, nausea) were noticed in 30% of patients. Authors concluded that although imatinib mesylate showed good tolerance profile, it failed to show satisfactory clinical responses in that group of patients [431]. A gynecologic oncology group enrolled 56 patients into a II phase clinical trial on the efficacy of imatinib mesylate monotherapy in recurrent or persistent epithelial ovarian or primary peritoneal carcinoma. The median PFS and OS observed were 2 and 16 months, respectively. The most common serious grade 3/4 toxicities were neutropenia, skin rash, pain, and electrolyte disturbances. Most tumors expressed c-Kit or PDGFR, and a higher expression was associated with shorter survival. However, GOG summarizes that imatinib mesylate had minimal single-agent activity [466,467,468]. Another II phase clinical trial was devoted to test 13 patients with platinum-resistant low-grade serum OC (LGSC) who had received up to 4 platinum- and/or taxane-containing chemotherapy regimens. One patient presented with stable disease for 7 months, but no complete or partial responses were obtained. Similarly, as in a previously discussed trial, there was no correlation between the expression of c-Kit or PDGF receptor and the extent of the clinical response. The most common toxicities were fatigue (66%0, nausea (66%), and diarrhea (41%). Despite good tolerance, imatinib mesylate has no activity in patients with platinum- and taxane-resistant LGSC [467].

### 9.6. DNMT1 Inhibitors

Enzyme DNA (cytosine-5)-methyltransferase 1 (DNMT1) catalyzes the transfer of methyl groups to specific CpG structures in DNA. Aberrant methylation has been observed in several tumors. The inhibition of this enzyme could potentially augment the tumor response against standard chemotherapy. The II phase clinical trial on DNMT1 inhibitor decitabine combined with platinum-based chemotherapy was performed in the group of 29 patients with recurrent partially chemo-sensitive (relapse 6–12 months after first line chemotherapy) ovarian cancer. Responses according to the RECIST criteria were observed in 46% of patients in carboplatin monotherapy arm, and in 8% of patients in the carboplatin/decitabine arm. The authors concluded that the addition of decitabine appeared to reduce rather than increase the efficacy of carboplatin [443]. In another study, the combination of low-dose decitabine treatment followed by reduced-dose carboplatin/paclitaxel regimen or reduced-dose carboplatin/paclitaxel regimen combined with cytokine-induced killer cells were tested in the group of 52 patients with both platinum-resistant and sensitive tumors. The response rate (ORR) was 24% and 31%, respectively. The most common side effects were grade 1/2 nausea, anorexia, fatigue, neutropenia, and anemia. In the opinion of the authors, low-dose decitabine/paclitaxel/carboplatin regimen seemed to be effective especially in patients with platinum-resistant OC, and when combined with adoptive immunotherapy. Another DNMT1 inhibitor guadecitabine was tested in combination with carboplatin in platinum-resistant recurrent ovarian cancer, in a II phase multicenter randomized clinical trial. After the enrollment and randomization, 100 patients were eligible and assigned either into the arm with guadecitabine + carboplatin treatment (n = 51), or to the arm of treatment of choice (topotecan, doxorubicin, paclitaxel or gemcitabine) (n = 49). The median PFS was not significantly different between arms; however, the 6-month PFS rate was higher in the group with guadecitabine (37% vs. 11%). Neutropenia was the most common serious side effect in that group [439]. Azacitidine, the next DNMT1 inhibitor, was tested together with carboplatin in 30 platinum-refractory or resistant patients. Response rate ORR was 14% with four cases of complete/partial responses and 10 cases of disease stabilization. Patients with platinum resistance achieved an ORR of 22%, median PFS of 5.6 months and a median OS of 23 months. This was the first evidence that a hypomethylating agent may partially reverse platinum resistance in ovarian cancer [450].

### 9.7. Histone Deacetylase (HDAC) Inhibitors

Histone deacetylase (HDAC) is the class of enzymes that allow the histones to wrap the DNA more tightly. HDAC inhibitors induce the accumulation of acetylated histones and transcription factors that cause cell cycle arrest. Vorinostat (ZOLINZA^®®^) known as suberanilohydroxamic acid (SAHA) is one of the HDAC inhibitors approved by the FDA for the treatment of hematologic malignancies. In a multicenter phase II GOG clinical trial, vorinostat was tested in monotherapy in the group of 27 platinum-refractory and resistant patients. Only two women achieved PFS of 6 months. The grade 3/4 toxicity was reported and included neutropenia, pain, thrombocytopenia, and neurologic symptoms. Vorinostat was found to be well tolerated but with minimal activity in monotherapy in that group of patients [452]. In another study, the combination of vorinostat with carboplatin and gemcitabine was tested in 15 patients with relapsed OC. Six patients had a partial response for the treatment regimen; however, the observed hematological toxicity was a serious obstacle for treatment continuation [453]. Similarly, serious toxicity was observed in the group of 18 patients with advanced OC subjected to combined adjuvant therapy with carboplatin, paclitaxel, and vorinostat in the first line chemotherapy. Although 39% of patients demonstrated complete response and the ORR was 50%, the serious toxicity forced the closure of the study. Except for grade 3/4 neutropenia and thrombocytopenia observed in 56% and 12% of patients, respectively, the most troublesome side effect was bowel anastomotic perforation in 17% of patients [454]. Belinostat is a hydroxamic acid-type HDAC inhibitor inducing apoptosis and sensitizing tumor cells for chemotherapy. Its activity as monotherapy was tested in a phase II clinical study in 18 and 14 patients with metastatic or recurrent platinum-resistant EOC and micropapillary ovarian tumors, respectively. The best response in both groups was the stabilization of the disease noticed in 9 (EOC) and 10 (micropapillary) tumors. The most serious side effect was thrombosis in 17% of patients [455]. In another study, belinostat was tested in combined therapy with carboplatin/paclitaxel in 35 women with recurrent advanced OC and platinum refractoriness or resistance. The ORR was 43% with 3 complete and 12 partial responses. When considering the platinum sensitivity, the ORR was 44% among resistant and 63% among sensitive patients, respectively. The most common adverse effects were nausea (83%), fatigue (74%), vomiting (63%), alopecia (57%), and diarrhea (37%). This study brought evidence that belinostat in combination with standard chemotherapy is well tolerated and effective in pretreated OC patients [456]. Entinostat is a benzamide derivative of HDAC, selectively inhibiting class I and IV HDAC. The II phase clinical trial on the efficacy of combined therapy using entinostat and anti-programmed death ligand-1 (anti-PD-L1) monoclonal antibody avelumab, compared to avelumab alone, was performed in 126 OC patients that had progression or recurred after the first-line chemotherapy. The ORR of both regimens did not differ, and was low (5–6%). Observed toxicity of combined therapy was higher compared to avelumab monotherapy and consisted of fatigue (46%), nausea (31%), diarrhea (26%), anemia (26%), and chills (20%). In conclusion, the entinostat–avelumab combined therapy was not sufficiently effective, and unfortunately showed increased toxicity [468].

### 9.8. Focal Adhesion Kinase (FAK) Inhibitor

Potentially interesting other approaches to OCSC therapy could be treatment focused on the disruption of pro-cancerous signals from the tumor microenvironment to OCSCs or the elimination of OCSCs by targeted immunotherapy with modified T or NK cells. Defactinib is an inhibitor of focal adhesion kinase (FAK) which contributes to the interplay between OCSCs and stromal cells of their niche. The II phase NCT01778803 clinical trial tested the efficacy of defactinib/paclitaxel combination in the treatment of 18 patients with refractory or advanced OC. Three patients presented with complete, partial response, and stabilization of the disease, respectively. The profile of toxicity was acceptable with grade 3 side effects, which included neutropenia, hyperbilirubinemia, thrombocytopenia, and anemia [469].

### 9.9. Immune Elimination of OCSCs

#### 9.9.1. Cancer Vaccine

Therapeutic approaches to the immune elimination of OCSCs are advanced on both a preclinical and clinical level. One of the proposed treatments is a cancer therapeutic vaccine. In a murine model, the vaccine constructed from SKOV3 CD117+CD44+ OCSCs cells was effective against xenografted tumors and reduced the CD117+CD44+ALDH1+ OCSC population [470].

#### 9.9.2. Monoclonal Antibodies

Another approach is to target OCSCs using monoclonal antibodies (moAb). An example of such therapy is treatment with catumaxomab (anti EpCAM moAb), ipilimumab (anti-CTLA-4 moAb), avelumab, pembrolizumab, durvalumab (anti-PD-L1 moAbs), and nivolumab (anti-PD-1 moAb), although these have not been originally developed as OCSC-specific monoclonal antibodies. Catumaxomab has been approved for treatment of malignant ascites in patients with EpCAM-positive OC where standard therapy is not available or no longer feasible [471]. The results of the clinical trial indicated that samples of ascites obtained from patients treated with catumaxomab showed the elimination of CD133+EpCAM+ OCSCs cells, and an increase in CD8+ and CD4+ effector T cell populations when compared to samples of untreated patients [472]. Ipilimumab, avelumab, pembrolizumab, durvalumab and nivolumab are the moAbs inhibiting immune checkpoint molecules or their ligands. They are also not OCSC-specific, as the expression of immune checkpoint molecules is not an exclusive feature of cancer stem cells. However, as was described earlier, OCSCs—similarly to other cancer cells—are able to use PD-1/PD-L1-dependent mechanisms to escape from the host immunosurveillance. There are plenty of finished and ongoing phase II/III clinical trials of immune checkpoint inhibitors either in monotherapy or in combination with chemotherapy, or with different targeted therapies. However, these clinical trials evaluating PD-1/PD-L1 inhibition in patients with advanced or relapsed OC have been disappointing (reviewed in: [473]). PD-L1 tumor expression and high tumor mutational load are usually used as predictive biomarkers for efficacy of immune checkpoint inhibitors. However, most high-grade OCs demonstrate less than 5% positivity for PD-L1 expression; moreover, expression is mostly evident on the immune cell infiltrates rather than on the tumor cells themselves. Additionally, high-grade OC is not a type of tumor characterized by high mutational load, and instead it has a copy number of alterations. The exceptions are endometroid and clear-cell tumors associated with mismatch repair defects (MMRds), which result in the accumulation of point mutations, and therefore have improved sensitivity to immune checkpoint inhibitors [474,475]. Conclusively, OCSCs failed in being the effective target for this group of drugs.

#### 9.9.3. CAR-T and CAR-NK Cells

Chimeric antigen receptors (CAR) are recombinant receptors constructed for the enhancement of T cell- or NK cell-mediated responses against specific tumor antigens. The first-generation of CAR-dependent adoptive immunotherapy targeting α-folate receptor (FR- α) was tested in a clinical setting, but without recordable effects [476]. The third-generation EpCAM-CAR-T cells were able to exhibit in vitro killing activity against OC cell line SKOV3, as well as significantly reduce the tumor size in OC xenograft mouse models. The anti-tumor activity of EpCAM-CAR-T cells against OC in vitro and in vivo indicated that the CAR-T might provide a promising therapeutic approach to OC [99]. The third-generation anti-CD133+ CAR engineered NK cells showed the specific elimination of CD133+ OCSCs in ovarian cancer cell lines and OC-cultured cells isolated from ascites. The sequential CAR-NK and cisplatin treatment showed the strongest killing effect [477]. Another third-generation anti CD44+ CAR engineered NK cells showed strong cytotoxic activity against CD44+ ovarian cancer SKOV3 and OVCAR3 cell lines and primary ovarian cancer cells harvested from ascites, compared to CD44-negative A2780 cells. The anti-cancer efficacy was further augmented by the simultaneous use of cisplatin treatment. Although the results of those two studies are promising, we should remember that preclinical studies are usually performed as experiments on pure cell lines or tumor-xenografted immunodeficient mice. These are idealized conditions which are not mimicked in clinical settings, where patients’ immune statuses and the effects of TIME inside tumors are far from simple. Experience indicates that the optimistic results of preclinical trials are usually not followed by similarly satisfactory clinical results. Therefore, although efficient in many cancers, CAR-T therapy in OC is still unsatisfactory, probably due to specific OCSC microenvironments in the peritoneal cavity. CAR-T cells against mucin 16 and mesothelin are being studied in ongoing clinical trials [478].

## 10. Conclusions and Future Perspectives

The results for the management of ovarian cancer are not satisfactory, despite the extensive surgical effort to obtain radical macroscopic cytoreduction, standard chemotherapy using platinum- and taxane-based drugs, and approved targeted therapy using bevacizumab and PARP inhibitors. Compared to the efficacy of treatment that has been obtained during the last decade in breast cancer, which was considered another female “killer tumor”, there is still great work to do in ovarian cancer patients. So, why is ovarian cancer (especially that of a high-grade serous type II tumor) so aggressive and insusceptible to therapy? In our opinion, there are several clues concerning this issue. Two of them are the most important, in our opinion. The first problem is the complexity and specificity of the tumor microenvironment. Ovarian cancer originates and propagates inside the peritoneal cavity. The peritoneal metastases (implants) emerge early in the course of the disease and represent the main method of tumor spread and recurrence. Adipocytes of the omentum, bowel appendices, and mesentery constitute the unique environment (cancer-associated adipocytes) transferring signals for differentiation and keeping OCSCs up, as well as being the source of metabolic and energetic support for cancer cells. Other important constituents of the tumor microenvironment are cancer-associated fibroblasts, mesenchymal stem cells, mesothelial cells, and immune cells, which together orchestrate to support OC growth and immune escape. Ascites which is an indispensable component of peritoneal environment in advanced cancer, is another specific modulator of OCSC growth. It provides secretory stimuli, is a medium to transport exosomes, and is a source of mechano-sensory signals to OCSCs. Finally, hypoxia, acidosis, and inflammation support OC tumorigenicity and aggressiveness. The second problem is the genetic profile of the most malignant tumors, including a very complex epigenetic regulation of tumor cells, including OCSCs. These two features, together with hypoxic/acidic and the inflammatory environment, are responsible for primary platinum refractoriness. Together with the signals from OCSC niches, these factors contribute to secondary platinum resistance, as well as to exclusive plasticity, heterogeneity, and the dynamics of OCSCs. Environment, genome, and epigenetic regulation make anti-OCSC therapy of ovarian cancer a challenging task. OCSCs exhibit phenotypic changes during all stages of tumor progression. The populations of OCSCs may differ in the same patient or among different patients depending on the cancer histologic type, advancement of the disease, patient health status and treatment regime. The recurrent disease may also be distinct from the primary disease due to specific molecular changes that occur during the process of disease recurrence. Moreover, the abundance of regulatory and signaling pathways in OCSCs’ disposition make simultaneous treatment with even two or three drugs (directed against different target molecules) ineffective, causing the tumor to escape from pharmacologic surveillance (analogically to escape from host immune surveillance). Therefore, clinical trials testing anti-OCSC activity of different drugs have not shown satisfactory efficacy so far, either in monotherapy or in combination with other anti-cancer drugs. What we need is the identification of markers for pharmacologic compliance or resistance and the stratification of patients in order to predict the sensitivity of OCSCs to different drugs, and to individualize therapy. In order to restore pharmacologic surveillance, samples of primary and secondary tumors (both peritoneal implants and recurrent tumors) should be sampled repetitively for characterization of a genetic profile and microenvironmental features of the tumor changing in the course of the disease, and individualized therapy should be applied which fits the actual status of the tumor and the patient. The hit against OCSCs should be always accompanied with the hit against its microenvironment and the potentialization of the host anti-tumor immune response. The disruption of inflammatory and hypoxic intraperitoneal conditions should further restrict tumor growth. Chemotherapy could accompany or alternatively follow such OCSC-targeted therapy. Different types of OC will probably need different approaches to the treatment. Similarly, different phases of the disease will probably also need diversification of the therapy. We propose to name such an approach to OC therapy as “Dynamic PHarmacologic survEillaNCE” (DEPHENCE approach). The aim of the DEPHENCE approach would be either to cure the patient, or more probably to control and stabilize the disease for a more acceptable and satisfactory time than it is possible nowadays. Such treatment would probably also help to avoid the increased toxicity of combined drug regimens, as many drugs indicate activity against mechanisms commonly met inside both cancer and healthy cells. Until this happens, we prognose that anti-OCSC therapy will hardly achieve moderate efficacy.

Another major problem to be solved in OC is to recognize the disease as soon as possible. Oncological treatment is more effective in early-recognized tumors. In ovarian cancer, 75% of patients are diagnosed in an advanced stage of the disease. To improve this, we need the effective populational identification of patients at risk of ovarian cancer as well as effective screening programs for this tumor. An identification of BRCA mutations or familial risk of ovarian cancer is an example of the first strategy which should be extended to newly recognized risk factors. Screening programs for the general female population are still lacking, although tumor markers, risk algorithms, and ultrasound examinations have improved early OC diagnoses in patients with adnexal masses. Despite evolving treatment modalities, we should concentrate on the improvement of early diagnostic tools. The identification of ovarian cancer-prone women on the basis of genome profiling or the search for single-tumor CTC cells or DNA in peripheral blood could improve diagnostic capability and make ovarian cancer more curable. Finally, taking into consideration that fallopian tube obstruction seems to be a protective factor against ovarian cancer, it is reasonable to search for potential infective factors responsible for triggering tubal/ovarian carcinogenesis. The identification of such an infective factor could pave the way for antimicrobial therapy (analogical to Helicobacter eradication) or vaccination (analogical to anti-HPV vaccines) as preventive techniques against ovarian cancer. All these activities should be incorporated into the DEPHENCE approach.

## Figures and Tables

**Table 2:** Examples of therapy directed against OCSCs including drugs tested in both experimental and clinical settings.

Target	Drug	Mechanism of Action	Clinical Trial	Reference
**Inhibition of Signaling Pathways**				
Wnt signaling pathway	Ipafricept (OMP54F28)	Inhibition of Fc-Frizzled 8 receptor	NCT02050178 Ia/Ib phase	[416]
	WNT974	Selective inhibitor of porcupine acetyltransferase (PORCN)—decreases Wnt secretion and lowers binding of Wnt to its receptor	Experimental	[417]
Hedgehog signaling pathway	Cyclopamine	Decrease in spheroid formation	Experimental	[169]
	Vismodegib (GDC-0449)	Smoothened (SMO) antagonist	NCT00739661 II phase	[418]
	Sonidegib (LDE225)	Smoothened (SMO) antagonist	NCT02195973 II phase	[116]
NOTCH signaling pathway	LY900009	Inhibitor of Υ-secretase protein	I phase	[419]
	MK-0752	Inhibitor of Υ-secretase protein	I phase	[420]
	Crenigascestat (LY3039478)	Inhibitor of Υ-secretase protein	Experimental	[421]
	RO4929097	Inhibitor of Υ-secretase protein	II phase	[422]
	Enoticumab (REGN421)	moAb against delta-like ligand-4 (DLL4)	I phase	[423]
	Demcizumab (OMP-21M18)	moAb against delta-like ligand-4 (DLL4)	SIERRA Ib phase	[424]
	Navicixizumab (OMP-305B83)	Dual moAb against DDL4 and VEGF	Ib phase	[425]
MAPK signaling pathway	Salinomycin	Polyether antibiotic—inhibitor of ABC-transporter system	Experimental	[426,427]
PI3K mTOR ERK/STAT3 signaling pathways	Metformin	Activation of AMP-activated protein kinase (AMPK) followed by the inhibition of signaling pathways and reduction in energy consumption by OCSCs	Experimental Observation of outcome in metformin users with OC NCT01579812 II phase	[428,429]
YAP/TAZ pathway	Verteporfin (Visudyn)	Second-generation photosensitizer—upon exposure to light of particular wavelength releases singlet oxygen and ROS toxic for cancer cells	Experimental	[430]
**Targeting OCSCs markers**				
CD117+	Imatinib mesylate (Gleevec)	Inhibition of tyrosine protein kinase KIT CD117) and platelet-derived growth factor-regulated pathway	NCT00510653 II phase	[431]
CD44+CD117+	Salinomycin + paclitaxel	Inhibitor of ABC-transporter system and chemotherapeutic	Experimental	[432]
CD133+	dCD133KDEL	Deimmunized pseudomonas exotoxin fused to anti-CD133 moAB inhibits OC growth	Experimental	[433]
ALDH1A+	673A	ALH1A inhibitor causes the accumulation of toxic aldehydes	Experimental	[434]
	CM37	ALH1A inhibitor causes the inhibition of cell spheroids and the down-regulation of OCT4 and SOX2	Experimental	[435]
CD44+MyD88+	NV-128	Isoflavone derivative—causes depression of mitochondrial function	Experimental	[436]
CD44v7/8+	CAR-T * ScFv-CD8-CD3ξ receptor	Increased cytotoxicity	I phase	[437]
EpCAM+	CAR-T ScFv-CD8-CD28-4IBB-CD3ξ receptor	Increased cytotoxicity	I phase	[438]
CD133+	CAR-T ScFv-CD28-4IBB-CD3ξ receptor	Increased cytotoxicity	I phase	[439]
**Drugs interfering with epigenetic regulation**				
DNA methylation	Decitabine	Inhibition of DNA-(cytosine-5)-methyltransferase-1 (DNMT1) in CAAs and stromal progenitor cells	Experimental	[440,441]
	Decitabine + carboplatin	Inhibitor of DNMT1 + chemotherapeutic	NCT01799083 II phase	[442,443,444,445,446]
	Decitabine + liposomal doxorubicin	Inhibitor of DNMT1 + chemotherapeutic	NCT00887796 I phase	[447]
	Guadecitabine + carboplatin	Inhibitor of DNMT1 + chemotherapeutic	NCT01696032 II phase RT	[448]
	Azacitidine	Inhibition of DNMT1 and increase in M1 type TAMs	Experimental	[449]
	Azacitidine + carboplatin	Inhibitor of DNMT1 + chemotherapeutic	NCT00529022 II phase	[450]
Histone deacetylation	Spiruchostatin A OBP-801/YM753	Histone deacetylase (HDAC) inhibitor—induces cell cycle arrest and apoptosis	Experimental	[451]
	Vorinostat	Suberanilohydroxamic—HDAC inhibitor induces accumulation of acetylated histones and transcription factors that cause cell cycle arrest	NCT00132067 II phase	[452]
	Vorinostat + carboplatin	HDAC inhibitor + chemotherapeutic	NCT00910000 NCT00976183 I phase	[453,454]
	Belinostat (PXD-101, Beleodaq)	Hydroxamic acid-type HDAC inhibitor inducing apoptosis and sensitizing tumor cells for chemotherapeutic	NCT00993616 II phase	[455]
	Belinostat + carboplatin	HDAC inhibitor + chemotherapeutic	NCT00421889 II phase	[456]
	Entinostat (MS-275)	Benzamide derivative of HDAC—selectively inhibits class I and IV HDAC	NCT02915523 II phase	[445]
**Other mechanisms of inhibition of OCSCs**				
OCSCs	Rexinoid (9cUAB30)	Synthetic composition of retinoid + receptor agonist—inhibition of proliferation and stimulation of apoptosis of OCSCs	Experimental	[457]
Focal adhesion kinase (FAK)	PF-271	ATP-competitive inhibitor of FAK activity prevents anchorage-independent OC growth	Experimental	[458]
	Defactinib (VS-6063)	Disruption of FAK Y^397^ residue and inhibition of PI3K/AKT signaling	Experimental NCT01778803 I phase	[428,459]
Src kinase	Saracatinib (AZD0530)	Src family kinase inhibitor—inhibits proliferation and induces apoptosis	Experimental	[460]
MEK	Selumetinib (AZD6244)	Inhibitor of mitogen-activated protein kinase kinase —inhibits proliferation and induces apoptosis	Experimental	[460]
Fatty acid synthase (FASN)	TVB-2640 + paclitaxel	Inhibitor of FASN	NCT02223247 I phase	[461]

* RT—randomized trial; CAR-T—chimeric antigen receptor T cells.

## Data Availability

Not applicable.
